# Multiple Regulatory Mechanisms Control the Production of CmrRST, an Atypical Signal Transduction System in Clostridioides difficile

**DOI:** 10.1128/mbio.02969-21

**Published:** 2022-02-15

**Authors:** Elizabeth M. Garrett, Anchal Mehra, Ognjen Sekulovic, Rita Tamayo

**Affiliations:** a Department of Microbiology and Immunology, University of North Carolina Chapel Hill, Chapel Hill, North Carolina, USA; b Department of Molecular Biology and Microbiology, Tufts University School of Medicine, Boston, Massachusetts, USA; University of Oklahoma Health Sciences Center

**Keywords:** phase variation, phenotypic heterogeneity, bet-hedging, cyclic diguanylate, c-di-GMP, motility, biofilm, population heterogeneity

## Abstract

Clostridioides difficile, an intestinal pathogen and leading cause of nosocomial infection, exhibits extensive phenotypic heterogeneity through phase variation. The signal transduction system CmrRST, which encodes two response regulators (CmrR and CmrT) and a sensor kinase (CmrS), impacts C. difficile cell and colony morphology, surface and swimming motility, biofilm formation, and virulence in an animal model. CmrRST is subject to phase variation through site-specific recombination and reversible inversion of the “*cmr* switch,” and expression of *cmrRST* is also regulated by cyclic diguanylate (c-di-GMP) through a riboswitch. The goal of this study was to determine how the *cmr* switch and c-di-GMP work together to regulate *cmrRST* expression. We generated “phase-locked” strains by mutating key residues in the right inverted repeat flanking the *cmr* switch. Phenotypic characterization of these phase-locked *cmr*-ON and -OFF strains demonstrates that they cannot switch between rough and smooth colony morphologies, respectively, or other CmrRST-associated phenotypes. Manipulation of c-di-GMP levels in these mutants showed that c-di-GMP promotes *cmrRST* expression and associated phenotypes independently of *cmr* switch orientation. We identified multiple promoters controlling *cmrRST* transcription, including one within the ON orientation of the *cmr* switch and another that is positively autoregulated by CmrR. Overall, this work reveals a complex regulatory network that governs *cmrRST* expression and a unique intersection of phase variation and c-di-GMP signaling. These findings suggest that multiple environmental signals impact the production of this signaling transduction system.

## INTRODUCTION

The ability to adapt to environmental changes is critical to bacterial survival, including that of pathogens, which can face rapidly changing conditions and stresses during infection (1–4). Sense-and-respond adaptation strategies often involve two-component systems (TCS) that consist of a sensor kinase and a cognate response regulator. In response to an activating signal, such as binding of a ligand or a change in pH, the sensor kinase autophosphorylates and activates the response regulator through transfer of the phosphoryl group (5, 6). Response regulators have a wide range of functions, and many control transcription through direct binding of DNA. The resulting transcriptional changes contribute to adaptation of the bacterium to the environmental stimulus sensed by the sensor kinase. Bacteria can also adapt to stimuli via intracellular small molecules such as cyclic diguanylate (c-di-GMP) (7, 8). The intracellular level of c-di-GMP is modulated by the opposing activities of diguanylate cyclases and phosphodiesterases that synthesize and degrade c-di-GMP, respectively; the production and function of these enzymes is controlled by environmental signals (9, 10). C-di-GMP is then recognized by specific protein or RNA receptors (riboswitches) that mediate the adaptive response (11–13).

In contrast, diversification of phenotypes in a bacterial population serves as a bet-hedging strategy to help ensure survival of the population as a whole. The development of phenotypically distinct variants, independent of environmental conditions, improves the odds that a subpopulation survives a sudden stress (2, 14). Phase variation, a mechanism of generating phenotypic heterogeneity, occurs through reversible genetic changes that typically cause an ON/OFF phenotypic “switch” (15, 16). Several mechanisms of phase variation have been described, including conservative site-specific recombination, in which a sequence-specific recombinase binds inverted repeats and mediates inversion of the intervening DNA (15, 17). The invertible DNA element contains regulatory information, such as a promoter, that impacts the expression of adjacent genes. In a well-characterized example in Escherichia coli, phase variation of fimbria production is mediated by the *fimS* invertible element, which contains a promoter that drives transcription of the fimbrial genes when properly oriented (18–20).

Clostridioides difficile is an intestinal pathogen and a leading cause of nosocomial infections in the United States. C. difficile infection (CDI) can result in mild to severe diarrhea and potentially fatal complications such as pseudomembranous colitis, toxic megacolon, and sepsis. Recent work has shown that C. difficile contains multiple invertible DNA elements flanked by inverted repeats, indicating a considerable capacity for phenotypic heterogeneity through phase variation (21, 22). Four of these invertible elements have been demonstrated to regulate downstream genes and related phenotypes in a phase variable manner. The Cdi4 invertible element, also called the flagellar switch, modulates expression of the *flgB* flagellar operon, resulting in phase variation of flagella (23, 24). This operon encodes the sigma factor SigD, which promotes the transcription of flagellar genes as well as transcription of *tcdR*, which encodes a direct activator of the C. difficile toxin genes *tcdA* and *tcdB* (25, 26). Accordingly, the production of these toxins is also phase variable (23, 24, 27). The Cdi1 invertible element mediates phase variation of CwpV, which contributes to phage resistance (28–31). The Cdi2 element, or *pdcB* switch, modulates the production of a c-di-GMP phosphodiesterase and affects intracellular c-di-GMP and related phenotypes (32, 33).

The Cdi6 invertible element, here called the “*cmr* switch,” regulates the expression of *cmrRST* in a phase-variable manner (21, 34). The *cmrRST* operon encodes a putative noncanonical TCS with two DNA-binding response regulators (CmrR and CmrT) and a sensor kinase (CmrS) (34). Both CmrR and CmrT contain phosphoreceiver and DNA binding domains, suggesting that they function as transcriptional regulators. Phase variation of CmrRST allows C. difficile to switch between rough and smooth colony morphologies that differ in several physiological characteristics. Through unknown mechanisms, CmrRST positively regulates type IV pilus-independent surface motility and cell elongation, and it negatively regulates swimming motility and biofilm formation. Furthermore, a *cmrR* mutant strain is deficient for colonization and shows attenuated virulence in a hamster model of infection, indicating a role for this regulatory system in CDI.

**FIG 1:**
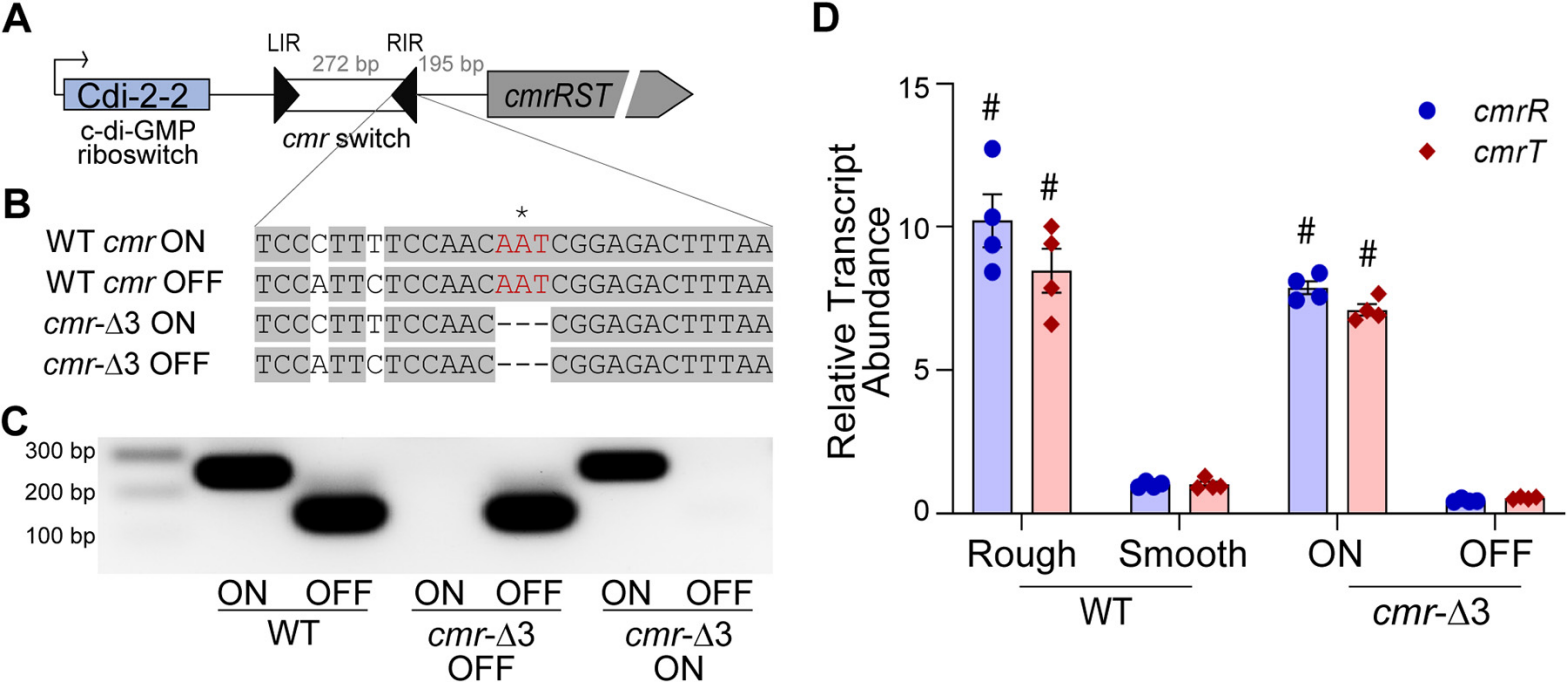
Generation of genetically phase-locked strains. (A) Diagram of the *cmrRST* locus. The previously identified c-di-GMP riboswitch Cdi-2-2 and putative associated promoter are indicated. Black triangles represent the inverted repeats flanking the invertible DNA sequence (*cmr* switch). The OFF orientation corresponds to the sequence present in the R20291 reference genome (GenBank accession number FN545816); the ON orientation corresponds to the inverse of that sequence. (B) Sequence of the right inverted repeat (RIR) of the *cmr* switch. Gray shading indicates sequence identity between the imperfect inverted repeats of the RIR in each orientation. Three nucleotides (shown in red) were deleted from the RIR to lock the *cmr* switch in the ON and OFF orientations. The asterisk (*) indicates the nucleotide at the site of recombination. (C) Orientation-specific PCR to detect each orientation of the *cmr* switch in WT, *cmr*-Δ3 OFF, and *cmr*-Δ3 ON strains. (D) qRT-PCR analysis for the *cmrR* and *cmrT* transcripts in WT rough and smooth isolates, *cmr*-Δ3 OFF, and *cmr*-Δ3 ON. Data from four biological replicates were analyzed using the ΔΔ*CT* method with *rpoC* as the reference gene and normalization to the WT smooth samples. Shown are means and standard error. #, *P* < 0.0001; two-way analysis of variance (ANOVA) with Sidak’s multiple-comparison posttest compared to the WT/smooth condition.

C-di-GMP riboswitches are widespread in the C. difficile genome and appear to be the primary mechanism of c-di-GMP regulation in this species (11, 12, 35, 36). Upstream of the *cmr* switch lies a class II c-di-GMP riboswitch that positively regulates *cmrRST* transcription in response to c-di-GMP (Fig. 1A) (35, 36). Increasing c-di-GMP levels results in the formation of the rough colony morphology, consistent with increased *cmrRST* expression (34). The relative contributions of the c-di-GMP riboswitch and the *cmr* switch to controlling expression of *cmrRST*, and therefore to the phenotypes controlled by this system, are unknown.

One challenge to the study of phase variation is its stochastic nature, which adds uncontrolled variation into an otherwise controlled experiment. Phase-locked strains in which the invertible element is prevented from inverting can be a useful tool with which to study phase-variable systems (23, 37). Our previous work on CmrRST relied primarily on characterization of wild-type (WT) C. difficile rough and smooth colony isolates, which have a strong bias for the ON and OFF *cmr* switch orientations, respectively, but remain capable of switch inversion and phenotypic switching (34). We aimed to characterize *cmrRST* regulation through the interplay of c-di-GMP and the *cmr* switch by generating phase-locked *cmr*-OFF and *cmr*-ON mutants. The results of this study indicate that *cmrRST* expression is subject to complex regulation by both sense-and-respond mechanisms and phase variation, highlighting the potential importance of this system to C. difficile physiology through the variety of activating signals.

## RESULTS

### Generation and phenotypic characterization of phase-locked *cmr*-ON and *cmr*-OFF strains.

To characterize the dual regulation of the *cmrRST* system by phase variation and c-di-GMP, we first generated phase-locked strains with the *cmr* switch fixed in the ON or OFF orientation. Phase locking can be achieved in multiple ways. One strategy is to delete the site-specific recombinase responsible for inversion. In C. difficile R20291, the RecV recombinase is required for inversion of the *cmr* switch (21). However, RecV mediates the inversion of multiple sequences; thus, a mutation in *recV* has pleiotropic effects (21, 23, 28). Instead, we chose to phase-lock the *cmr* switch by mutating an inverted repeat sequence to prevent site-specific recombination from occurring (38). The exact nucleotides at which recombination and inversion of the *cmr* switch occur were previously identified (21). We used allelic exchange to delete the nucleotide at the site of inversion in the right inverted repeat (RIR) (position 3,736,177) in the R20291 genome, as well as one additional nucleotide on each side (Fig. 1B). Prior work determined that mutating the equivalent residues in the flagellar switch RIR eliminated switch inversion, resulting in phase-locked strains (39). We recovered independent mutants with the *cmr* switch in either orientation and designated them *cmr*-Δ3 ON and *cmr-*Δ3 OFF. These mutants were subjected to orientation-specific PCR to confirm that they are genotypically phase-locked. In contrast to the WT parent that yielded both *cmr*-ON and *cmr*-OFF products indicating heterogeneity in *cmr* switch orientation in that population of cells, *cmr*-Δ3 ON exclusively yielded a PCR product corresponding to the ON orientation, and *cmr*-Δ3 OFF only yielded the OFF orientation product (Fig. 1C).

Previous work showed that R20291 rough and smooth colony variants correlate with *cmr*-ON and *cmr*-OFF gene expression and phenotypes, respectively (34). Specifically, the *cmr*-ON, rough colony variant exhibits greater surface growth and reduced swimming motility than the *cmr*-OFF, smooth colony variant. Furthermore, ectopic expression of *cmrR* and *cmrT* individually stimulated rough colony formation and surface motility while inhibiting flagellum-mediated swimming motility, although only *cmrT* was required for rough colony development and surface motility. To conclusively determine the effect of *cmr* switch orientation on gene expression, we measured the abundance of the *cmrR* and *cmrT* transcripts in the *cmr*-Δ3 ON and *cmr*-Δ3 OFF mutants. Nonlocked rough and smooth isolates were included as controls (34). Both *cmrR* and *cmrT* transcripts were 7- to 10-fold more abundant in C. difficile with the *cmr* switch in the ON orientation, whether in the naturally arising rough colony variant or in *cmr*-Δ3 ON (Fig. 1D). Therefore, the three nucleotides at the site of RecV-mediated recombination are required for *cmr* switch inversion, and the ON orientation corresponding to the inverse of the published R20291 genome promotes expression of *cmrRST.*

**FIG 2:**
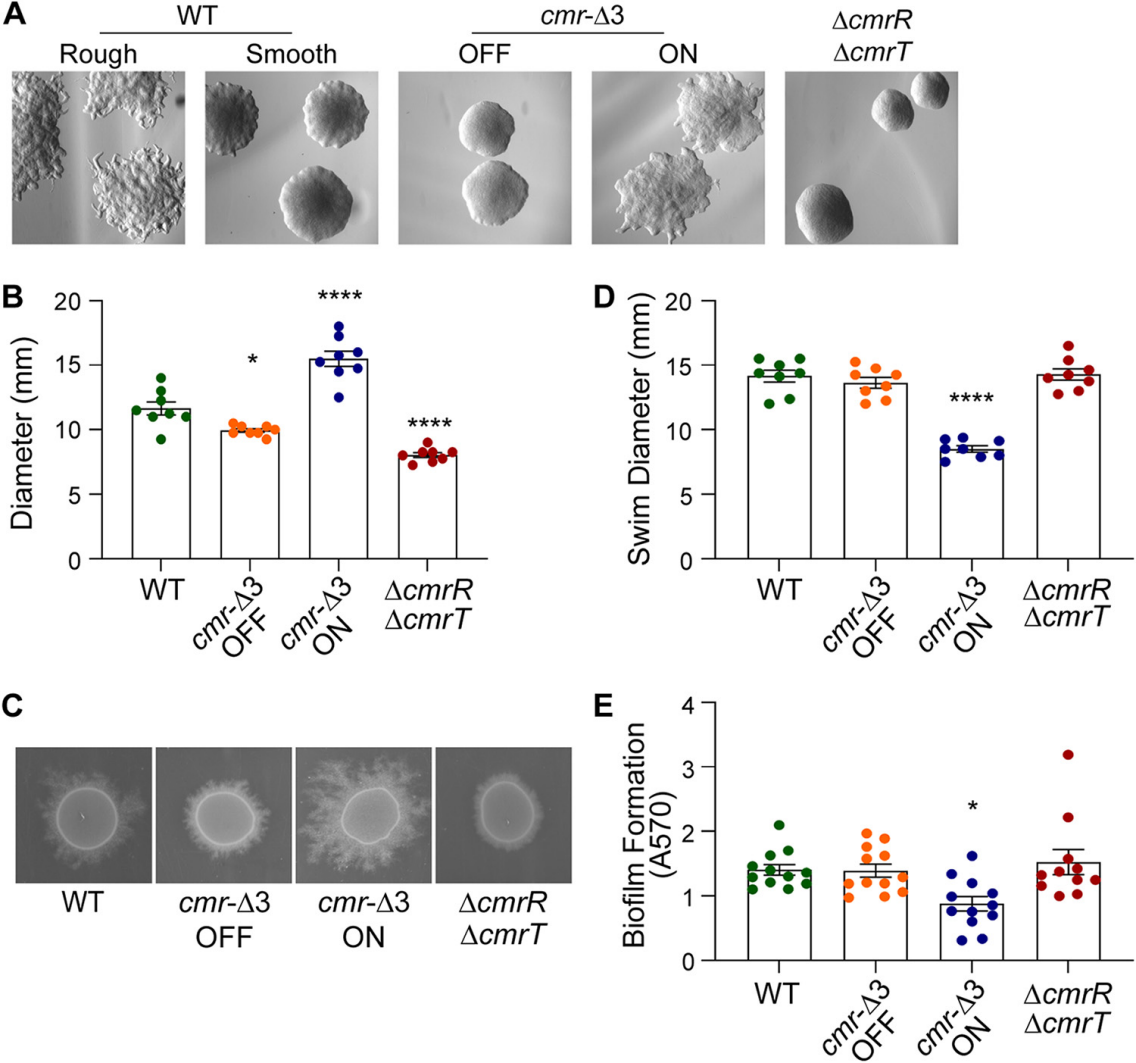
Mutating the RIR of the *cmr* switch results in phenotypically phase-locked strains. (A) Colony morphology of WT rough and smooth isolates, *cmr*-Δ3 OFF, *cmr*-Δ3 ON, and Δ*cmrR*Δ*cmrT*. Shown are representative images after 24 h of growth on BHIS agar. (B) Surface motility of WT, *cmr*-Δ3 OFF, *cmr*-Δ3 ON, and Δ*cmrR*Δ*cmrT* quantified by measuring the diameter of growth after 72 h. (C) Representative images of surface motility after 72 h of growth. (D) Swimming motility of WT, *cmr*-Δ3 OFF, *cmr*-Δ3 ON, and the Δ*cmrR*Δ*cmrT* mutant quantified by measuring the diameter of growth in 0.5× BHIS 0.3% agar after 48 h. (E) Biofilm formation after 24 h in BHIS-1% glucose-50 mM sodium phosphate, quantified by crystal violet staining. (B, D, and E) Shown are means and standard error, with symbols representing independent samples from at least 2 independent experiments. *, *P* < 0.05; ****, *P* < 0.0001; one-way ANOVA with Dunnett’s multiple-comparison posttest compared to the WT.

The *cmr*-Δ3 phase-locked mutants were then evaluated for the phenotypes previously associated with *cmrRST* expression. As a control we included WT R20291, which is capable of CmrRST phase variation and generation of both rough and smooth colonies, as well as a Δ*cmrR* Δ*cmrT* double mutant that forms only smooth colonies (Fig. 2A). The *cmr*-Δ3 OFF mutant formed exclusively smooth colonies, similar to Δ*cmrR* Δ*cmrT*. *cmr*-Δ3 ON formed rough colonies that resemble those in the WT, though they appeared to have less defined topology (Fig. 2A). Also consistent with increased *cmrRST* expression, *cmr*-Δ3 ON displayed greater surface motility than the WT, while *cmr*-Δ3 OFF had decreased surface motility more similar to that of Δ*cmrR* Δ*cmrT* (Fig. 2B and C). Finally, *cmr*-Δ3 ON showed decreased swimming motility and biofilm formation compared to all other strains (Fig. 2D and E). Together, these results reinforce the model that the *cmr* switch orientation modulates expression of *cmrRST* and the assignment of the respective ON and OFF switch orientations.

### C-di-GMP regulates *cmrRST* expression independently of the *cmr* switch.

**FIG 3:**
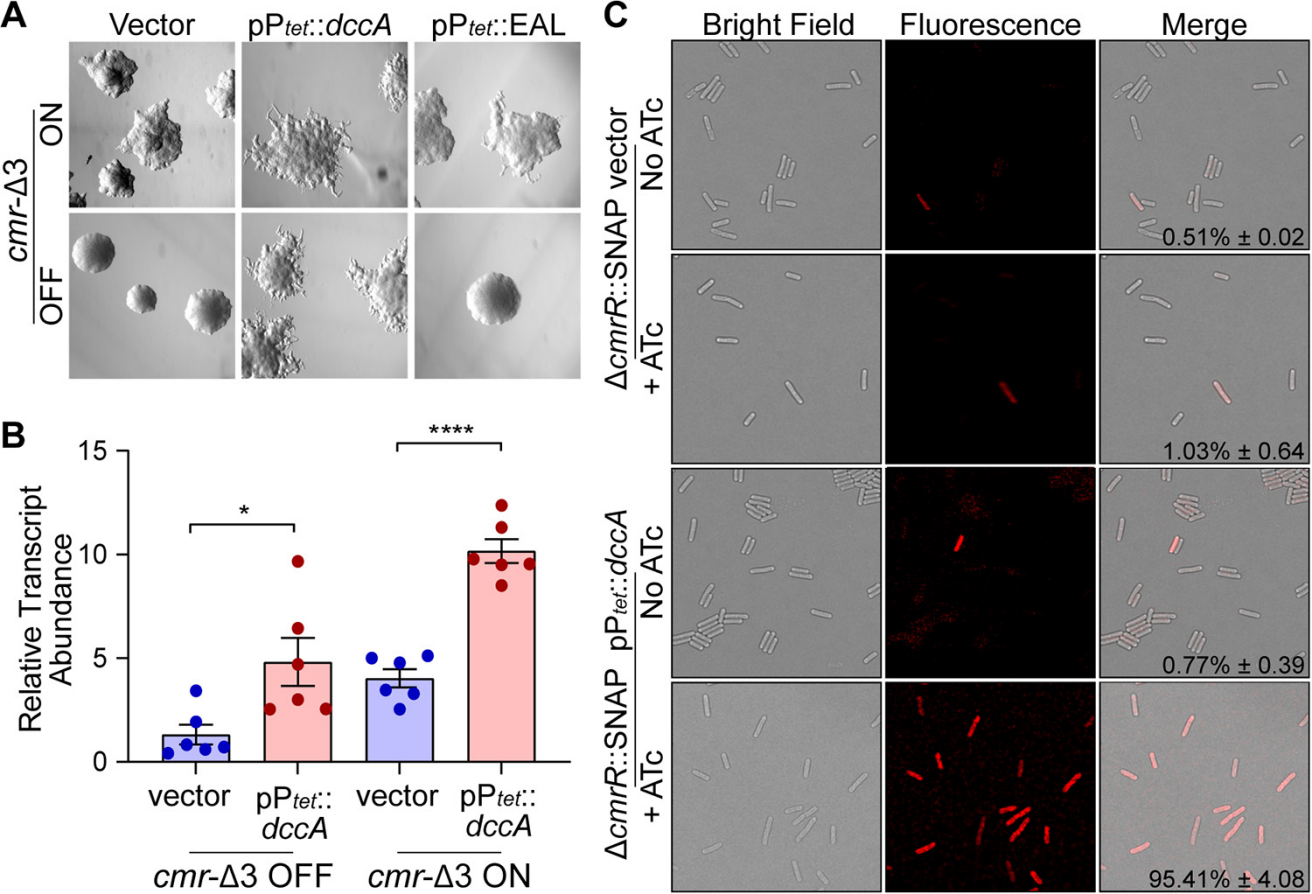
Regulation of *cmrRST* by c-di-GMP occurs independently of *cmr* switch orientation. (A) Colony morphology of *cmr*-Δ3 OFF and *cmr*-Δ3 ON containing either vector, pP*_tet_*::*dccA*, or pP*_tet_*::EAL. Strains were grown on BHIS-agar with 20 ng/mL ATc to induce DGC and EAL gene expression. (B) qRT-PCR analysis of *cmrS* transcript abundance in *cmr*-Δ3 OFF and *cmr*-Δ3 ON strains carrying pP*_tet_*::*dccA* or vector control grown in BHIS broth with 20 ng/mL ATc. Data were analyzed using the ΔΔ*CT* method with *rpoC* as the reference gene and normalization to *cmr*-Δ3 OFF with vector. Shown are means with standard error of six biological replicates from two independent experiments. *, *P* < 0.05; ****, *P* < 0.0001; one-way ANOVA with Tukey’s multiple-comparison posttest. (C) Representative images of Δ*cmrR*::SNAP carrying pP*_tet_*::*dccA* or vector control grown in BHIS broth with 20 ng/mL ATc. Samples were imaged at ×60 magnification by light and fluorescence microscopy following staining with SNAP-Cell TMR-Star substrate. Percentages of total cells that were fluorescence-positive were determined from at least three fields each from two biological replicates. Shown are means and standard deviations.

Upstream of the *cmr* switch is a c-di-GMP riboswitch sequence, adding another regulatory layer to the transcription of *cmrRST* (35, 36). The two regulatory features could act independently with one regulatory element dominant over the other, or they might act coordinately such that both the riboswitch and *cmr* switch must be in the “on” state to allow *cmrRST* transcription. To distinguish between these possibilities, we modulated c-di-GMP levels in the phase-locked *cmr*-Δ3 ON and OFF strains by overexpressing the diguanylate cyclase *dccA* (pP*_tet_*::*dccA*) to increase intracellular c-di-GMP or the catalytic EAL domain of the phosphodiesterase *pdcA*, which hydrolyzes c-di-GMP, to reduce c-di-GMP (pP*_tet_*::EAL) (10, 40, 41). Increasing c-di-GMP through overexpression of *dccA* resulted in rough colony formation in *cmr*-Δ3 OFF, which normally forms smooth colonies (Fig. 3A). Additionally, increasing c-di-GMP enhanced the rough colony appearance of *cmr*-Δ3 ON. In contrast, reduction of c-di-GMP through overexpression of the EAL domain did not visibly affect the morphology of *cmr*-Δ3 ON or OFF, suggesting that the impact of EAL overexpression on c-di-GMP levels was insufficient to alter expression of *cmrRST* compared to the WT with vector under these conditions. Overexpression of *dccA* led to a significant 2- to 3-fold increase in the abundance of the *cmrS* transcript, which served as a marker for *cmrRST* expression, in both *cmr*-Δ3 ON and OFF, consistent with the colony morphology phenotypes observed (Fig. 3B).

These methods assess changes in *cmrRST* expression as a population average. To determine the effects of the c-di-GMP riboswitch and the *cmr* switch on the heterogeneity of *cmrRST* expression among individual bacteria, we used a strain in which *cmrR* was replaced by a codon-optimized SNAP-tag gene reporter, Δ*cmrR*::SNAP (21). The pP*_tet_*::*dccA* plasmid and vector control were introduced into this strain. In Δ*cmrR*::SNAP with vector and in the uninduced control, approximately 1% of the population fluoresced (Fig. 3C). This result is similar to those of prior analyses showing that a minority of WT cells express *cmrRST* (21). Induction of *dccA* increased fluorescence-positive cells to >95%. This result was not due to a shift in *cmr* switch orientation; by quantitative PCR (qPCR), the orientation of the *cmr* switch was not significantly different between WT overexpressing *dccA* and the vector control (Fig. S1). Therefore, the higher percentage of fluorescent cells reflects *cmrRST* expression in response to c-di-GMP. Together, these data indicate that expression of *cmrRST* from the upstream promoter and c-di-GMP riboswitch is not dependent on the orientation of the *cmr* switch. Rather, high c-di-GMP levels increase *cmrRST* expression regardless of *cmr* switch orientation.

10.1128/mBio.02969-21.1 FIG S1 C-di-GMP does not cause inversion of the *cmr* switch. Δ*cmrR*::SNAP carrying pP*_tet_*::*dccA* or vector control was grown to the mid-exponential phase in BHIS broth with or without 20 ng/mL ATc. Genomic DNA (gDNA) was collected for qPCR analysis of *cmr* switch orientation. Data are expressed as the percent OFF orientation. Shown are the means and standard deviations of six biological replicates from two independent experiments. No significant differences; two-way ANOVA with Tukey’s multiple-comparison test. Download FIG S1, PDF file, 0.1 MB. Copyright © 2022 Garrett et al. 2022 Garrett et al. https://creativecommons.org/licenses/by/4.0/ This content is distributed under the terms of the Creative Commons Attribution 4.0 International license.

### The *cmr* switch contains a promoter in the ON orientation.

**FIG 4:**
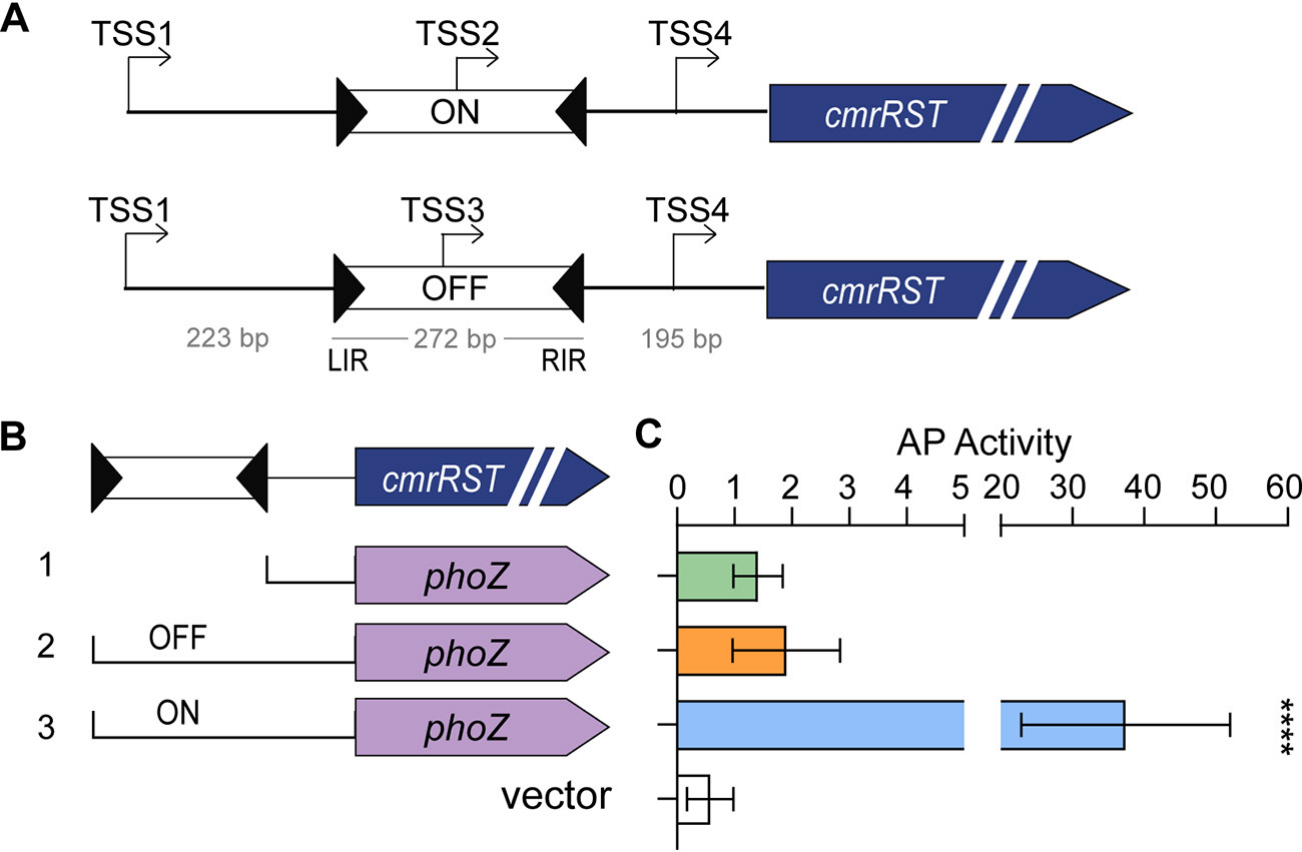
Mapping of transcriptional start sites (TSS) upstream of *cmrRST*. (A) Diagram of TSS identified by 5′ RACE using RNA derived from *cmr*-ON and *cmr*-OFF templates. (B) Alkaline phosphatase reporters used to detect promoter activity. Brackets indicate the regions from the native *cmr* locus that were used to make transcriptional reporters. Construct 1 contains the region between the 3′ end of the right inverted repeat and the 5′ end of *cmrR* coding sequence (pMC123::TSS4-*phoZ*). Construct 2 includes the region in construct 1 plus the *cmr* switch in the OFF orientation (pMC123::*cmr*OFF/TSS4-*phoZ*). Construct 3 is identical to construct 2 but with the *cmr* switch in the ON orientation (pMC123::*cmr*ON/TSS4-*phoZ*). (C) Alkaline phosphatase (AP) activity of *C. difficile* with reporters in panel B. Shown are the means and standard deviations from 12 biological replicates from 4 independent experiments. ****, *P* < 0.0001; one-way ANOVA with Dunnett’s multiple-comparison test.

The best-characterized phase variation regulatory mechanisms involve a promoter encoded within the invertible element that drives expression of adjacent genes when in the proper orientation (20). Our data suggest that *cmrRST* transcription originates from multiple sites in a manner dependent on internal c-di-GMP concentrations and the orientation of the *cmr* switch. Therefore, we sought to map the transcriptional start sites (TSS) in strains reflecting these different states. Instead of using *cmr*-Δ3 phase-locked strains, which are missing three nucleotides of unknown significance to transcription, we used strains in which the *cmr* switch is locked by a mutation in *recV*, designated *recV cmr*-ON and *recV cmr*-OFF (Fig. S2). Additionally, *dccA* was overexpressed in *recV cmr*-OFF to identify any TSS associated with increased c-di-GMP and transcriptional readthrough via the riboswitch. Using 5′ rapid amplification of cDNA ends (RACE), four total TSS were identified in *recV cmr*-ON, *recV cmr*-OFF, and *recV cmr*-OFF pDccA. In all strains, a TSS designated TSS1 was identified 699 nucleotides (nt) upstream of the *cmrR* start codon and located upstream of the c-di-GMP riboswitch (Fig. 4A, Fig. S3). Another TSS, designated TSS2, was found in *recV cmr*-ON, located 336 nt upstream of *cmrR* and mapping within the *cmr* switch in the ON orientation. A third TSS, designated TSS3, was identified 342 nt upstream of the *cmrR* start codon in both *recV cmr*-OFF strains. This position maps TSS3 to within the *cmr* switch, appearing only in transcripts from the *cmr*-OFF strains. In all three strains, a fourth TSS (TSS4) was identified 90 nt upstream from the *cmrR* start codon.

10.1128/mBio.02969-21.2 FIG S2 The *cmr* switch is phase-locked in *recV*-deficient strains. Orientation-specific PCR to detect each orientation of the *cmr* switch in WT, *recV cmr-*OFF, and *recV cmr-*ON. Download FIG S2, PDF file, 0.1 MB. Copyright © 2022 Garrett et al. 2022 Garrett et al. https://creativecommons.org/licenses/by/4.0/ This content is distributed under the terms of the Creative Commons Attribution 4.0 International license.

10.1128/mBio.02969-21.3 FIG S3 Identification and mapping of multiple transcriptional start sites upstream of *cmrRST*. Map of TSS identified by 5′ RACE in *recV cmr*-ON, *recV cmr*-OFF, and *recV cmr*-OFF pDccA. Gray highlight indicates the Cdi-2-2 riboswitch sequence. Red and blue text denote the OFF and ON orientations of the *cmr* switch, respectively. Bold text indicates the inverted repeats. Green highlights mark TSS. Underlined text indicates putative –10/–35 sites. Yellow highlight denotes the *cmrR* start codon. Download FIG S3, PDF file, 0.3 MB. Copyright © 2022 Garrett et al. 2022 Garrett et al. https://creativecommons.org/licenses/by/4.0/ This content is distributed under the terms of the Creative Commons Attribution 4.0 International license.

The 5′ RACE results confirm an expected TSS upstream of the c-di-GMP riboswitch and suggest that expression of *cmrRST* can originate from multiple additional sites. TSS2 within the *cmr* switch in the ON orientation was identified; however, we unexpectedly identified TSS3 within the *cmr* switch in the OFF orientation. To elucidate how phase-variable *cmrRST* expression is mediated, we examined the functionality of the newly identified TSS. We created a series of transcriptional reporter fusions of the alkaline phosphatase (AP) gene *phoZ* to regions immediately upstream of *cmrRST* (Fig. 4B) (42). Two of the fusions contain the upstream region from the left inverted repeat (LIR) to the start of the *cmrR* coding sequence. These fusions differ in the orientation of the invertible element; ON (pMC123::*cmr*ON/TSS4-*phoZ*) encompasses TSS2, and OFF (pMC123::*cmr*OFF/TSS4-*phoZ*) encompasses TSS3. Both constructs also include TSS4. Additionally, we included a reporter with the region from the 3′ end of the RIR to the start of the *cmrR* coding sequence (pMC123::TSS4-*phoZ*), which contains TSS4, as well as a promoterless control (vector). To prevent inversion of the *cmr* switch present in the reporters once in C. difficile, we assayed AP activity in a *recV* mutant lacking the recombinase necessary for inversion (21). C. difficile with pMC123::TSS4-*phoZ* (Fig. 4B, construct 1) or pMC123::*cmr*OFF/TSS4-*phoZ* (construct 2) showed no significant difference in activity compared to the promoterless control (Fig. 4C). In contrast, C. difficile with pMC123::*cmr*ON/TSS4-*phoZ* (construct 3) exhibited 20-fold higher AP activity (Fig. 4C). These results indicate the presence of a functional promoter when the *cmr* switch is in the ON orientation but not the OFF orientation.

### CmrR positively autoregulates expression of *cmrRST*.

**FIG 5:**
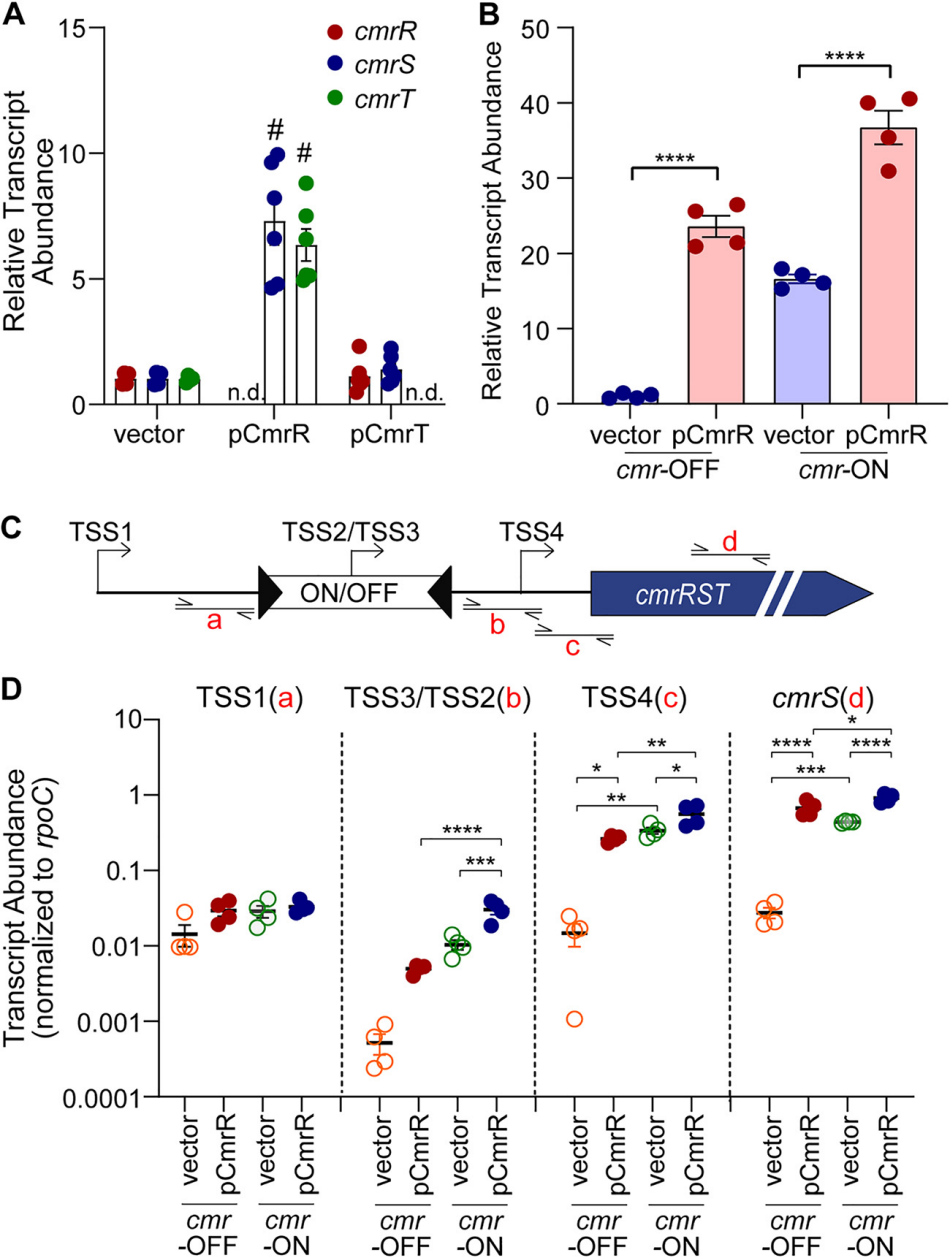
CmrR positively regulates *cmrRST* expression. qRT-PCR measurement of *cmrRST* transcript abundance. Strains were grown with ATc induction (10 ng/mL for vector and pCmrR, 2 ng/mL for pCmrT) prior to RNA isolation. (A) WT with pCmrR, pCmrT, and vector control. n.d., not determined; *cmrR* and *cmrT* were not measured in strains overexpressing those genes. #, *P* < 0.0001; unpaired *t* test. (B) qRT-PCR analysis of *cmrS* transcript abundance of *recV cmr*-OFF and *cmr-*ON, each with pCmrR or vector control. The *recV cmr*-OFF with vector strain serves as the reference condition for ΔΔ*CT* analysis. Shown are means and standard deviations of 4 biological replicates. (C) Diagram showing regions of transcript measured in panel D. (D) qRT-PCR analysis of *cmrRST* transcripts with values normalized to *rpoC*. (B and D) Shown are means and standard error from independent replicates. *, *P* < 0.05; **, *P* < 0.01; ***, *P* < 0.001; ****, *P* < 0.0001; one-way ANOVA with Tukey’s multiple-comparison test.

Response regulators often have the property of autoregulating their expression (43). To determine whether CmrR and/or CmrT have the ability to transcriptionally regulate *cmrRST*, we overexpressed *cmrR* (pCmrR) and *cmrT* (pCmrT) in C. difficile and analyzed *cmrRST* expression by quantitative reverse transcriptase PCR (qRT-PCR). Overexpression of *cmrR* resulted in a significant 7-fold increase in transcript abundance of *cmrS* and *cmrT* compared to the vector control (Fig. 5A). In contrast, overexpression of *cmrT* had no effect on *cmrR* or *cmrS* transcript abundance. These results indicate that CmrR, but not CmrT, transcriptionally regulates the *cmrRST* operon.

To assess whether CmrR-mediated regulation is dependent on the orientation of the invertible element, we overexpressed *cmrR* from a plasmid in the phase-locked *recV cmr*-ON and *cmr*-OFF mutants. We then used qRT-PCR measuring *cmrS* to assess expression. Overexpression of *cmrR* significantly increased *cmrS* transcript abundance compared to the respective vector control in both *recV cmr*-OFF and *cmr*-ON mutants (Fig. 5B). While overexpression of *cmrR* in *recV cmr*-OFF resulted in over a 20-fold increase in *cmrS* transcript abundance relative to vector control, the *cmrS* transcript increased only about 2-fold in *recV cmr-*ON pCmrR relative to the vector control.

We considered the possibility that CmrR regulates *cmrRST* expression from one of the previously identified TSS. To address this possibility, we used qRT-PCR to measure the transcript abundance by probing sequences immediately downstream of TSS1, the *cmr* switch containing TSS2 or TSS3, and TSS4, as well as within *cmrS* (Fig. 5C). Because transcript abundance differed substantially depending on the region measured, we expressed the data normalized to *rpoC* transcript abundance rather than the ΔΔ*CT* method. Overexpression of *cmrR* did not alter transcript abundance when probing immediately downstream of TSS1, regardless of *cmr* switch orientation, indicating that CmrR-mediated autoregulation occurs downstream of the TSS1 promoter (Fig. 5D). In contrast, when probing immediately downstream of the *cmr* switch (TSS2/TSS3), *cmrR* overexpression resulted in 11-fold higher transcript abundance in *recV cmr*-OFF relative to the vector control, though the difference did not reach statistical significance (Fig. 5D). Transcript abundance was 23-fold higher in *recV cmr*-ON than in *recV cmr*-OFF (vector controls, *P* < 0.01), and *cmrR* overexpression in the *cmr*-ON background led to a further 3-fold increase. Similar changes were observed when probing downstream of TSS4; *cmrR* overexpression increased transcript abundance 31-fold in *recV cmr*-OFF and 60-fold in *recV cmr*-ON compared to the *recV cmr*-OFF vector control (Fig. 5D). These results suggest that CmrR autoregulates *cmrRST* expression from the promoter corresponding to TSS2, TSS4, or both. Notably, transcript abundance was 29- and 36-fold higher when measuring downstream of TSS4 compared to TSS3 (*cmr*-OFF) and TSS2 (*cmr*-ON), respectively, indicating that the TSS2 promoter is weaker than the TSS4 promoter under these conditions.

### CmrR autoregulation of *cmrRST* expression requires the TSS4 promoter.

**FIG 6:**
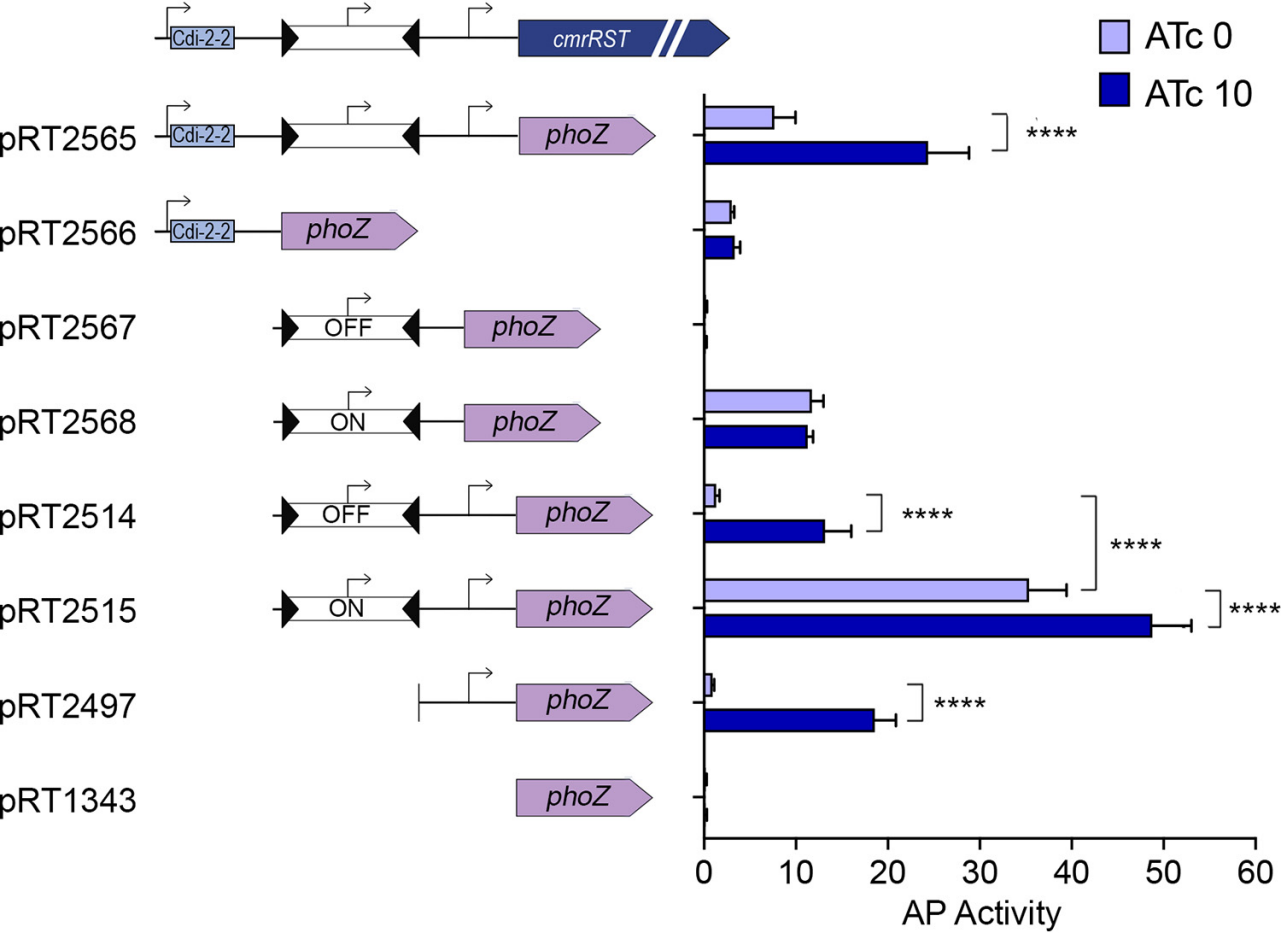
CmrR autoregulation occurs at the TSS4 promoter and is enhanced by the *cmr*-ON switch sequence. Alkaline phosphatase reporter activity for regions of the *cmr* 5′ untranslated region (UTR) containing one or more putative promoters in C. difficile with ATc-inducible P*_tet_*::*cmrR* at an ectopic site. (Left) Diagrams of the plasmid-borne reporter fusions carried by the strain. (Right) AP reporter activity after growth with or without 10 ng/mL ATc to induce *cmrR* expression (Fig. S4). Shown are means and standard deviations from three independent experiments. ****, *P* < 0.0001; two-way ANOVA and Sidak’s posttest.

To further define the promoter autoregulated by CmrR, we generated reporter strains in which different portions of the *cmrRST* regulatory region were transcriptionally fused to a *phoZ* (alkaline phosphatase, AP) reporter gene (42). We first constructed a strain in which *cmrR*, under the control of the P*_tet_* promoter, was inserted at an ectopic site on the RT1693 (*recV cmr-*OFF) chromosome. This strain allowed anhydrotetracycline (ATc)-inducible expression of *cmrR* (Fig. S4), prevented unintended inversion of the *cmr* switch (Fig. S2), and permitted the use of plasmid-borne *phoZ* reporters. Figure 6 shows the combinations of the TSS1/c-di-GMP riboswitch, the *cmr* switch orientation (TSS2/ON or TSS3/OFF), and the region encompassing TSS4.

10.1128/mBio.02969-21.4 FIG S4 Inducible expression of *cmrR* from an ectopic chromosomal site. R20291 *recV* CDR2492::P*_tet_*::*cmrR* was grown to the mid-exponential phase in BHIS medium with and without 10 ng/mL ATc to induce *cmrR* expression. qRT-PCR was used to measure *cmrR* transcript abundance using 3 different primer sets to distinguish *cmrR* expression from the native site (primers R3111/R3112), from the ectopic site (primers R3113/R3112), and total *cmrR* (primers R2298/R2299). Data are expressed as relative transcript abundance under the ATc condition compared to that without ATc, with normalization to *rpoC* as the reference strain. Induction with ATc resulted in a 9.0-fold increase in *cmrR* mRNA from the ectopic site and a 2.6-fold increase from the native *cmrRST* locus, with a cumulative 12.7-fold increase in *cmrR* transcript. Shown are means and standard error. *, *P* < 0.05; ***, *P* < 0.001; ****, *P* < 0.0001 by unpaired *t* test. Download FIG S4, PDF file, 0.1 MB. Copyright © 2022 Garrett et al. 2022 Garrett et al. https://creativecommons.org/licenses/by/4.0/ This content is distributed under the terms of the Creative Commons Attribution 4.0 International license.

The promoter corresponding to TSS1, located upstream the c-di-GMP riboswitch, displayed similar transcriptional activity regardless of ATc (pRT2566), indicating that CmrR does not autoregulate expression at this site. This result is consistent with the qRT-PCR analysis of the native *cmrRST* site (Fig. 5), and it further suggests that *cmrR* overexpression does not impact global intracellular c-di-GMP levels. The promoter corresponding to TSS3 (pRT2567), present in the *cmr*-OFF switch orientation, yielded no detectable AP activity when isolated from upstream (TSS1) and downstream (TSS4) sequences and was not affected by overexpression of *cmrR*. Adding the downstream sequence to the *cmr*-OFF switch region (TSS3 + TSS4, pRT2514) resulted in no AP activity in the absence of ATc but led to 10-fold increased AP activity when *cmrR* was overexpressed.

The construct containing only the *cmr*-ON switch sequence (TSS2, pRT2568) displayed comparable levels of transcriptional activity with and without *cmrR* overexpression. The addition of the downstream sequence (TSS2 + TSS4, pRT2515) yielded baseline AP activity 3-fold higher than the TSS2-only reporter and also conferred CmrR-mediated autoactivation. Finally, the reporter containing the region downstream of the *cmr* switch alone (TSS4, pRT2497) did not show AP activity in the absence of ATc but did exhibit 20-fold activation by *cmrR* overexpression. The baseline AP activity and degree of activation by CmrR was comparable to that from pRT2514 (TSS3 + TSS4), confirming that there is no active promoter in the *cmr* switch when in the OFF orientation under these conditions. Overall, only the constructs with the TSS4 region resulted in increased activity upon *cmrR* overexpression, indicating that the TSS4 promoter is required for CmrR-mediated autoregulation of *cmrRST*. Further, the highest transcriptional activity in the absence of *cmrR* overexpression was seen in the *cmr*-ON construct in which TSS2 and TSS4 are both present, suggesting additive effects of the promoters or the disruption of an important sequence in the constructs separating the two elements.

### *cmrRST* and its upstream regulatory elements are well conserved in C. difficile.

C. difficile strains from multiple ribotypes form rough and smooth colonies associated with phase-variable *cmrRST* expression (34). To examine the potential scope of CmrRST phase variation and function in C. difficile, we used NCBI BLAST to determine the extent of conservation of *cmrRST* and its upstream regulatory region using R20291 as the reference sequence. The analysis examined the 895 bases 5′ of *cmrR*, which contains a c-di-GMP riboswitch and the *cmr* switch (Fig. 1A), and the *cmrRST* coding sequences. Among 71 C. difficile whole-genome sequences available in NCBI (taxonomic ID 1496), C. difficile strains shared high sequence identity to the reference for both the riboswitch (98.9% ± 1.2) and the *cmr* invertible element (98.1% ± 1.4) (Fig. S5). This similarity is comparable to the average percent sequence identity for the *cmrRST* operon (98.7% ± 1.1). These results indicate that the c-di-GMP riboswitch and invertible element upstream of *cmrRST* are highly conserved across many C. difficile strains from divergent ribotypes and underscores the importance of this operon and its regulation to C. difficile physiology.

10.1128/mBio.02969-21.5 FIG S5 *cmrRST* and upstream regulatory sequences are highly conserved across C. difficile strains and ribotypes. Heat map showing the percent sequence identity of 71 sequenced C. difficile strains in the NCBI database compared to the R20291 reference sequence (NCBI accession number FN545816.1). If the strain has been ribotyped, the ribotype is indicated on the left. For the *cmr* switch, both orientations were used as the reference, and the highest percent sequence identity is shown. The following nucleotides were used as the reference sequence from R20291: riboswitch, 3,736,512 to 3,736,863; *cmr* switch, 3,735,969 to 3,736,513; *cmrRST*, 3,732,474 to 3,735,968. Download FIG S5, PDF file, 0.2 MB. Copyright © 2022 Garrett et al. 2022 Garrett et al. https://creativecommons.org/licenses/by/4.0/ This content is distributed under the terms of the Creative Commons Attribution 4.0 International license.

## DISCUSSION

In this study, we demonstrate that multiple regulatory mechanisms control the transcription of *cmrRST*. Our results support a model in which, under basal c-di-GMP conditions, the orientation of the *cmr* switch determines the expression level of *cmrRST*. This phase variation mechanism involves the reversible inversion of a promoter within the *cmr* switch sequence. Increasing intracellular c-di-GMP augments *cmrRST* expression independent of *cmr* switch orientation. Our data also show that expression of *cmrRST* is also subject to autoregulation by CmrR, which occurs at an additional promoter downstream of the *cmr* switch and is enhanced by the orientation of the *cmr* switch. Therefore, multiple environmental signals may impact *cmrRST* expression through c-di-GMP signaling, phase variation, and the activation of the response regulator CmrR. CmrRST has important roles in C. difficile cell and colony morphology, motility, biofilm formation, and virulence, suggesting multiple contexts in which distinct environmental stimuli and selective pressures must be integrated to appropriately control *cmrRST* expression at the single-cell and population levels. This work represents the first account of a unique intersection of regulatory mechanisms controlling the expression of a signal transduction system that broadly impacts C. difficile physiology and disease development.

Phenotypic analysis of phase-locked R20291 mutants (*cmr*-Δ3 ON and *cmr*-Δ3 OFF) showed that they behave similarly to WT rough and smooth populations, respectively. Expression of *cmrRST* in the locked-ON mutant was equivalent to that of WT rough isolates, while expression in the *cmr*-Δ3 OFF mutant was similar to that of WT smooth isolates. Consistent with these results, *cmr*-Δ3 ON yielded rough colonies, increased surface motility, and decreased swimming motility and biofilm formation compared to WT smooth isolates, the *cmr*-OFF mutant, and the Δ*cmrR* Δ*cmrT* mutant. Interestingly, while *cmr*-Δ3 ON forms rough colonies, they are not identical to those formed in WT populations, and there is still some heterogeneity of colony morphology. This observation may reflect differences in activity of CmrR or CmrT or that other factors contribute to colony morphology. Other work has suggested heterogeneity in c-di-GMP levels among individual C. difficile cells (23, 44), so c-di-GMP may also result in colony morphology differences via modulation of *cmrRST* expression.

By artificially manipulating intracellular c-di-GMP levels in the *cmr* locked mutants, we found that c-di-GMP promotes *cmrRST* expression regardless of the orientation of the *cmr* switch. These findings are consistent with observed phenotypes; the *cmr*-Δ3 OFF mutant exhibited surface motility intermediate between that of the WT and the Δ*cmrR* Δ*cmrT* mutant. In the *cmr*-Δ3 OFF mutant, *cmrRST* could still be expressed from the TSS1 promoter upstream of the invertible element if c-di-GMP levels are sufficiently high. Intracellular c-di-GMP levels have been shown to increase in C. difficile with growth on a surface (41), which is consistent with c-di-GMP serving as a signal to enhance CmrRST-mediated surface motility regardless of *cmr* switch orientation.

**FIG 7:**
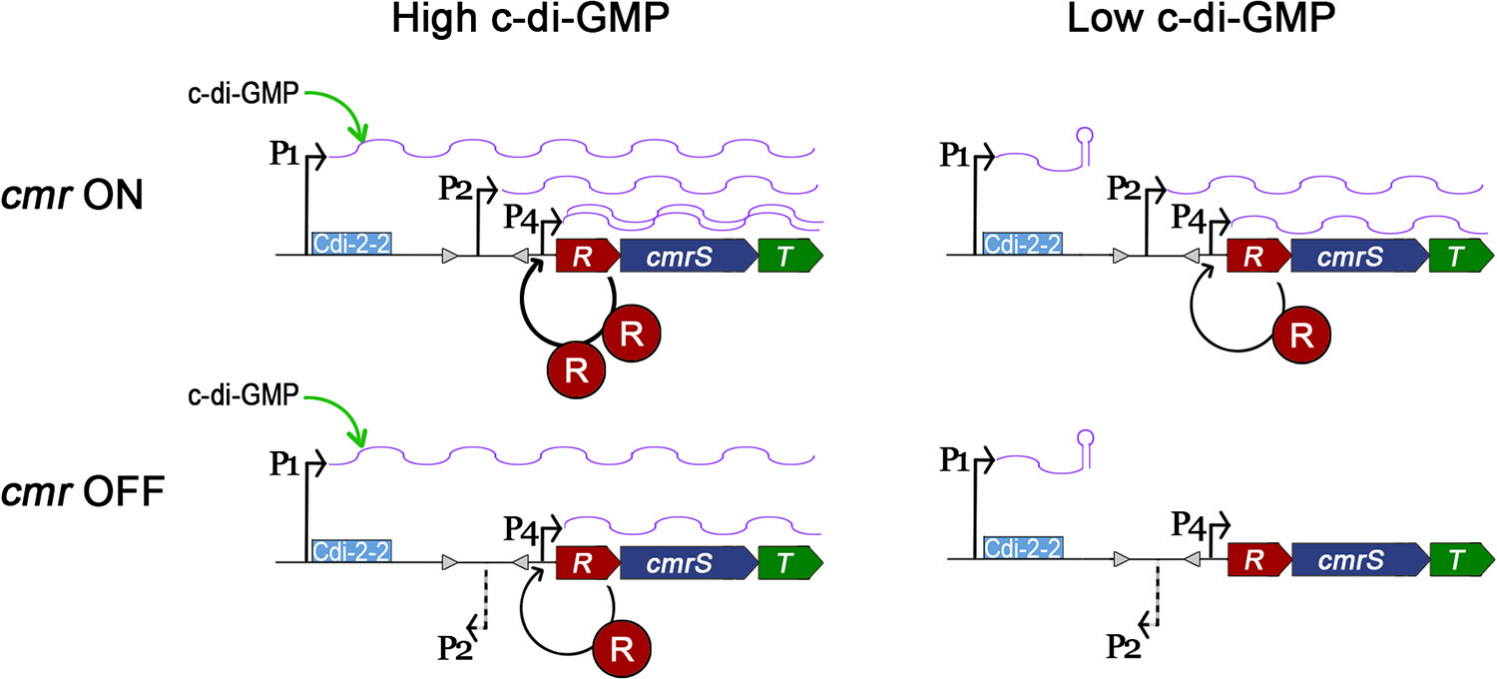
Model of multilevel regulation of *cmrRST* transcription. Three promoters control the expression of *cmrRST*. Promoter P1, corresponding to TSS1, generates an mRNA with a c-di-GMP riboswitch that is permissive for transcriptional readthrough when c-di-GMP is bound. The *cmr* switch contains a promoter (P2) corresponding the TSS2 when in the ON orientation. An additional promoter, P4, lies between the *cmr* switch and the *cmrRST* coding sequences. Transcription from P4 is positively regulated by CmrR. P1 and P2 independently regulate *cmrRST* transcription, while P4 activity is enhanced when the *cmr* switch is in the ON orientation.

Multiple TSS were identified upstream of *cmrRST* using 5′ RACE (Fig. S3). TSS1 mapped upstream of the c-di-GMP riboswitch sequence and is preceded by –10 and –35 sites. TSS2 was identified within the *cmr* switch in *cmr*-ON C. difficile. The TSS2 region showed transcriptional activity using the alkaline phosphatase reporter, and TSS2 is preceded by potential –10 and –35 sites. TSS3 was detected within the *cmr* switch in *cmr*-OFF bacteria; while qRT-PCR analysis suggested low-level transcription and potential responsiveness to *cmrR* overexpression, the TSS3 region lacked detectable transcriptional activity using the *phoZ* reporter assay, and no –10/–35 sites were identifiable. TSS4 was found between the *cmr* switch and the translational start of *cmrR* in both *cmr*-ON and *cmr*-OFF strains; the TSS4 region showed transcriptional activity when *cmrR* was overexpressed. A longer region containing both TSS2 and TSS4 showed maximal reporter activity, and the contributions of the TSS2 and TSS4 promoters to transcription appeared additive. Furthermore, only the reporters containing TSS4 exhibited CmrR-mediated autoactivation. Together, these results support a model (Fig. 7) in which the TSS1 promoter (P1) acts independently of the downstream promoters and yields an mRNA with a c-di-GMP riboswitch that enhances transcription readthrough when the c-di-GMP ligand is bound. The *cmr* switch in the ON state contains a promoter (P2) corresponding to TSS2, while the inverse orientation of the *cmr* switch lacks an active promoter. An additional promoter (P4) downstream of the *cmr* switch is activated by CmrR and autoactivates *cmrRST*. These regulatory elements are poised to modulate *cmrRST* expression in response to distinct environmental stimuli. Interestingly, CmrT does not regulate the expression of *cmrRST*, suggesting that CmrR and CmrT have different DNA specificities and, accordingly, distinct regulons. Future work will identify the genes directly and indirectly regulated by CmrR, CmrT, or both as well as determine their consensus DNA binding sites, which will elucidate the functions of these coexpressed response regulators.

In other bacteria, the reversible inversion of a promoter via site-specific DNA recombination is a common mechanism of phase-variable expression of adjacent genes (15, 45). Two additional mechanisms have been described in C. difficile. Phase variation of the cell wall protein CwpV in C. difficile occurs as a result of site-specific recombination of a sequence mapping to the 5′ untranslated region of *cwpV*. In one orientation, the invertible DNA sequence results in the formation of an intrinsic terminator in the mRNA, preventing expression of the downstream gene *cwpV*; the intrinsic terminator does not form in the mRNA with the sequence in the inverse orientation, allowing *cwpV* transcription to occur (28). Flagellar phase variation in C. difficile also occurs through an mRNA-mediated mechanism, where one orientation of the invertible sequence yields an mRNA permissive for transcriptional readthrough, while the other orientation results in Rho-mediated transcription termination (23, 24, 27, 44). Notably, the region upstream of the *flgB* operon has a similar arrangement to that of *cmrRST*, with a c-di-GMP riboswitch preceding the switch that undergoes site-specific recombination (23, 25, 36). However, the riboswitch upstream of *flgB* negatively regulates transcriptional readthrough, so binding of c-di-GMP by the riboswitch leads to transcription termination precluding synthesis of longer transcripts containing the flagellar switch regardless of its orientation. Thus, phase variation and c-di-GMP regulation of flagellar gene expression are linked, in contrast to the *cmrRST* system in which c-di-GMP and the *cmr* switch independently modulate expression.

In summary, this work demonstrates that *cmrRST* expression is subject to multilayered regulation with multiple potential inputs from environmental signals. The complexity of this regulatory network suggests that *cmrRST* expression, and therefore its transcriptional targets, requires careful control. Further work that defines the signals which promote *cmrRST* expression will provide important insights into the role of this TCS in C. difficile physiology and pathogenesis.

## MATERIALS AND METHODS

### Growth and maintenance of bacterial strains.

Table S1 lists the strains and plasmids used in this study. C. difficile R20291 and derivative strains were maintained in an anaerobic environment of 90% N_2_, 5% CO_2_, and 5% H_2_. C. difficile strains were grown statically at 37°C in brain heart infusion (BHI), BHIS (37 g/L Bacto brain heart infusion, 5 g/L yeast extract), or tryptone yeast (TY; 30 g/L Bacto tryptone, 20 g/L yeast extract, 1 g/L thioglycolate) medium as indicated. E. coli strains were grown in Luria Bertani medium at 37°C with aeration. Antibiotics were used where indicated at the following concentrations: chloramphenicol (Cm), 10 μg/mL; thiamphenicol (Tm), 10 μg/mL; kanamycin (Kan), 100 μg/mL; ampicillin (Amp), 100 μg/mL.

10.1128/mBio.02969-21.6 TABLE S1 Strains and plasmids used in this study. Download Table S1, PDF file, 0.2 MB. Copyright © 2022 Garrett et al. 2022 Garrett et al. https://creativecommons.org/licenses/by/4.0/ This content is distributed under the terms of the Creative Commons Attribution 4.0 International license.

### Construction of bacterial strains and plasmids.

Table S2 lists the primers used in this study. Detailed methods for the generation of plasmids and strains used in this study are provided in Text S1. Strain and plasmid information is listed in Table S1.

10.1128/mBio.02969-21.7 TABLE S2 Oligonucleotides used in this study. Download Table S2, PDF file, 0.1 MB. Copyright © 2022 Garrett et al. 2022 Garrett et al. https://creativecommons.org/licenses/by/4.0/ This content is distributed under the terms of the Creative Commons Attribution 4.0 International license.

10.1128/mBio.02969-21.8 TEXT S1 Supplemental methods used for this study. Download Text S1, PDF file, 0.2 MB. Copyright © 2022 Garrett et al. 2022 Garrett et al. https://creativecommons.org/licenses/by/4.0/ This content is distributed under the terms of the Creative Commons Attribution 4.0 International license.

### Quantitative reverse transcriptase PCR.

For analysis of *cmrR* and *cmrT* expression in C. difficile R20291 (WT), *cmr*-Δ3 ON, and *cmr*-Δ3 OFF, these strains were grown overnight (16 h) in TY medium, and 5 μL was spotted on BHIS-agar. After 24 h, growth was collected, suspended in 1:1 ethanol:acetone, and stored at −80°C for subsequent RNA isolation. For analysis of transcript abundance in R20291 strains carrying pCmrR, pCmrT, or vector, these strains were grown overnight in TY-Tm medium. Cultures were diluted 1:30 in BHIS-Tm broth. After 2 h of growth, ATc was added to induce gene expression (WT with vector or pCmrR, 10 ng/mL; WT with pCmrT, 2 ng/mL). Samples were collected at the mid-exponential phase and saved in 1:1 ethanol:acetone at −80°C.

RNA was extracted as described previously (46) and treated with a TURBO DNA-free kit (Life Technologies) to remove contaminating genomic DNA. cDNA was synthesized using the high-capacity cDNA reverse transcription kit (Applied Biosystems) using the manufacturer’s protocols (24, 40). Real-time PCR was performed using the SensiFAST SYBR and fluorescein kit (Bioline) as previously described (21, 34). Data were analyzed using *rpoC* as the reference gene. Primers are as follows: *rpoC*, R850/R851; *cmrR*, R2298/R2299; *cmrT*, R2537/R2538; *cmrS*, R2539/R2540; TSS1, R2745/R2746; TSS2/3, R2751/R2804; TSS4, R2803/R2716.

### Orientation-specific PCR.

Orientation-specific PCR was done as previously described (23, 34). Colonies grown on BHIS-agar were boiled to produce lysate for PCR using primers R2270/R2271 to detect the ON orientation (241- bp product) and R2271/R2272 to detect the OFF orientation (140-bp product). PCR products were separated on a tris-acetate-EDTA (TAE)-1.5% agarose gel.

### Quantification of switch orientation by quantitative PCR.

R20291 *cmrR*::SNAP pP*_tet_*::*dccA* (RT2500) and vector control (RT2501) were grown overnight in TY-Tm broth and then diluted 1:30 in BHIS-Tm containing 20 ng/mL ATc to induce *dccA* expression. After growth to the mid-exponential phase, genomic DNA was purified by phenol:chloroform:isopropanol extraction and ethanol precipitation. qPCR was performed as previously described with 100 ng of DNA per 20-μL reaction and 100 nM primers (34). Data were analyzed using the ΔΔ*CT* method, with *rpoA* as the reference gene and the indicated control conditions. The primers are as follows: ON orientation, R2270/R2271; OFF orientation, R2271/R2272; and *rpoA*, R2273/R2274.

### Microscopy.

To image whole colonies, strains were grown on BHIS-Tm agar for 24 h. For *cmr*-Δ3 ON/OFF with pP*_tet_*::*dccA*, pP*_tet_*::EAL, or vector, ATc (20 ng/mL) was included in the agar. Colonies were imaged using a Keyence BZ-X810 microscope or Zeiss Stereo Discovery V8 dissecting microscope with a glass stage.

Fluorescence microscopy of SNAP-labeled strains was performed as previously described (21). R20291 *cmrR*::SNAP pP*_tet_*::*dccA* or vector control was grown overnight in TY-Tm broth. Cultures were diluted 1:30 in BHIS-Tm. After 2 h of growth, ATc (20 ng/mL) was added for induction. Mid-exponential-phase samples were pelleted, washed, and incubated with SNAP-Cell TMR-Star (New England Biolabs) at 37°C for 30 min. Samples were further washed and mounted on 1% agarose pads. Imaging was done using a Keyence BZ-X810 microscope with an Olympus 100× PlanFluor objective. Bright-field and visualization of red fluorescence with a Chroma filter (ET545/30x, EM620/60m) was done using the same image capture settings for all samples. Cells were counted on at least three fields from two biological replicates for each strain and condition.

### Phenotype assays.

Motility and biofilm assays were done as previously described (34, 40); further details are provided in Text S1.

### Alkaline phosphatase assays.

Strains carrying *phoZ* reporters were grown in BHIS medium to an optical density at 600 nm (OD_600_) of ∼1.0, and then 1.5 mL of culture was collected, pelleted, and frozen at −20°C. Samples were thawed, and AP activity using the substrate *p*-nitrophenyl phosphate was measured as previously described (23, 42).

### 5′ rapid amplification of cDNA ends (RACE).

R20291 *recV cmr-*OFF (RT1693), *recV cmr-*OFF pDccA (RT2184), and *recV cmr-*ON (RT2520) were grown in BHIS to the mid-exponential phase. RT2184 was grown with Tm and induced with 1 μg/mL nisin (induces *dccA* expression from the P*_cpr_* promoter). Samples were collected and frozen in 1:1 ethanol:acetone. RNA was extracted and purified using the RNeasy minikit (Qiagen). cDNA was synthesized with the 5′ RACE system for rapid amplification of cDNA ends kit (Thermo Fisher) using the manufacturer’s protocol. Briefly, cDNA was synthesized using primer R2792 (for all strains) or R3171 (in *cmr*-ON only) and SuperScript II reverse transcriptase. The samples were treated with RNase mix and then column purified and treated with terminal deoxynucleotidyl transferase (TdT). The products were then PCR amplified with the provided anchor primer and primer R2793 (for all strains) or R3172 (in *cmr-*ON only) and then subjected to Sanger sequencing.
