# Supplementary material for: Multiple Regulatory Mechanisms Control the Production of CmrRST, an Atypical Signal Transduction System in Clostridioides difficile
Source: mBio. 2022 Feb 15;13(1):e02969-21. doi: 10.1128/mbio.02969-21 (PMC8844915; doi:10.1128/mbio.02969-21)
Supplement: FIG S1 [file mbio.02969-21-sf001.pdf]

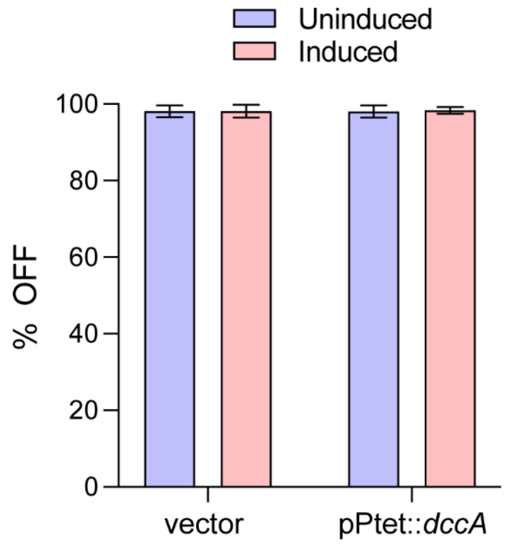

Fig S1. C-di-GMP does not cause inversion of the *cmr* switch. Δ*cmrR*::SNAP carrying pP$_{tet}$::*dccA* or vector control was grown to mid-exponential phase in BHIS broth with or without 20 ng/mL ATc. gDNA was collected for qPCR analysis of *cmr* switch orientation. Data are expressed as the percent OFF orientation. Shown are the means and standard deviations of six biological replicates from two independent experiments. No significant differences, two-way ANOVA with Tukey's multiple comparisons.
