# Supplementary material for: Multiple Regulatory Mechanisms Control the Production of CmrRST, an Atypical Signal Transduction System in Clostridioides difficile
Source: mBio. 2022 Feb 15;13(1):e02969-21. doi: 10.1128/mbio.02969-21 (PMC8844915; doi:10.1128/mbio.02969-21)
Supplement: FIG S2 [file mbio.02969-21-sf002.pdf]

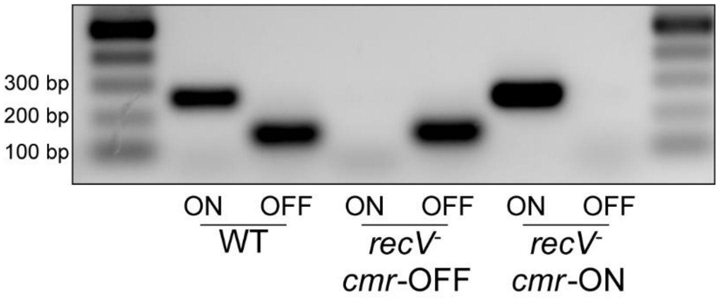

Fig S2. The *cmr* switch is phase-locked in *recV*-deficient strains. Orientation-specific PCR to detect the orientation of the *cmr* switch in WT, *recV cmr*-OFF, and *recV cmr*-ON.
