# Supplementary material for: Multiple Regulatory Mechanisms Control the Production of CmrRST, an Atypical Signal Transduction System in Clostridioides difficile
Source: mBio. 2022 Feb 15;13(1):e02969-21. doi: 10.1128/mbio.02969-21 (PMC8844915; doi:10.1128/mbio.02969-21)
Supplement: FIG S3 [file mbio.02969-21-sf003.pdf]

```
-35                    -10         TSS1
TTGACAAAATAATATTACAATGTTAGAATAAAAAAGAAATTTATGAAAATATATAAAAGATTTTTAATAAAAATATT

TTAGAAACTGAGAAGTATATCTTATTATTGGGCATCTGGAGATATATGGAGTTAGTGGTGCAACCGGCTATGAATAT

AATTTTTATATTGCATAGTCTTTTTTTGCTTTAAAATTAACAAAAATGTTGCAAAATTTGAAAATAAGAGTATTAGT

CGTTAAGATTTTTATTGATAGGTGAAATTTTGGCTTTTAAAGTAGCCATTTGTTGGAAAAGGGAAATTTTTTAAAAA
                                    TTAAAGTAGCCATTTGTTGGAGAATGGAGCTTAAAGTCATT

                                                                          -10
GTATAATACTATTATAATAAGAAAATAAAGTTTTTATAAACATTGAGATGCAAAATATAAACCTTTTGTAACAATAT
TTCACTTATAAATTTATTATTTTTCTAGAAACTTGTCTATTGGCTAGTTTGAGTATTGATTTATATGATTTATATGG
                                                        -35
       TSS3
ATTTACCAAAATAATACAATTTATTTTATATTATAACCATATAAATCATATAAATCAATACTCAAACTAGCCAATAG
TTATAATATAAAATAAATTGTATTATTTTGGTAAATATATTGTTACAAAAGGTTTATATTTTGCATCTCAATGTTTA
 -10          TSS2

ACAAGTTTCTAGAAAAATAATAAATTTATAAGTGAAAATGACTTTAAGCTCCATTCTCCAACAATCGGAGACTTTAA
TAAAAACTTTATTTTCTTATTATAATAGTATTATACTTTTTAAAAAATTTCCCTTTTCCAACAATCGGAGACTTTAA

                                                                      -35
TTAGTATTTAATAATAAATCTTAATGTTCAATACAATATTCAAGAATTGTTCAAAAATACTTAAGTATTATAGTCTA

                -10         TSS4
GTTTGTAAAGATATTATAAAATAATAATACTAGTTATATATGAAAATAAAACATTATAATTTGTAATAATTATAAAA

CTTGAGATTATGATTAAAATACCTTTGAATGGAGTGGTGTAATG
```

Fig S3. Identification and mapping of multiple transcriptional start sites upstream of *cmrRST*. Map of transcriptional start sites (TSS) identified by 5' RACE in *recV cmr*-ON, *recV cmr*-OFF and *recV cmr*-OFF pDccA. Gray highlight indicates the Cdi-2-2 riboswitch sequence. Red and blue text denote the OFF and ON orientations of the *cmr* switch, respectively. Bold text indicates the inverted repeats. Green highlights mark TSS. Underlined text indicates putative -10/-35 sites. Yellow highlight denotes the *cmrR* start codon.
