# Supplementary material for: Multiple Regulatory Mechanisms Control the Production of CmrRST, an Atypical Signal Transduction System in Clostridioides difficile
Source: mBio. 2022 Feb 15;13(1):e02969-21. doi: 10.1128/mbio.02969-21 (PMC8844915; doi:10.1128/mbio.02969-21)
Supplement: FIG S4 [file mbio.02969-21-sf004.pdf]

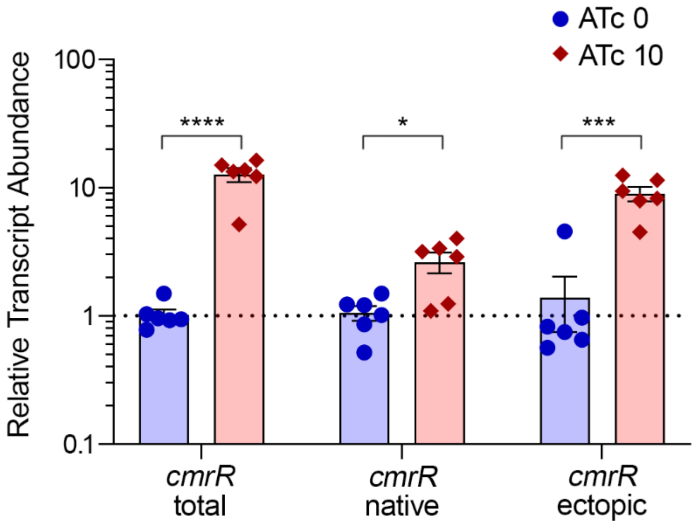

Fig S4. Inducible expression of *cmrR* from an ectopic chromosomal site. R20291 *recV* CDR2492::P$_{tet}$::*cmrR* was grown to mid-exponential phase in BHIS medium with and without 10 ng/mL ATc to induce *cmrR* expression. qRT-PCR was used to measure *cmrR* transcript abundance using 3 different primer sets to distinguish *cmrR* expression from the native site (primers R3111/R3112), from the ectopic site (primers R3113/R3112), and total *cmrR* (primers R2298/R2299). Data are expressed as relative transcript abundance in the ATc condition compared to that without ATc, with normalization to *rpoC* as the reference strain. Induction with ATc resulted in a 9.0-fold increase in *cmrR* mRNA from the ectopic site and a 2.6-fold increase from the native *cmrRST* locus, with a cumulative 12.7-fold increase in *cmrR* transcript. Shown are means and standard error. * $p < 0.05$, *** $p < 0.001$, **** $p < 0.0001$ by unpaired t-test
