# Supplementary material for: Multiple Regulatory Mechanisms Control the Production of CmrRST, an Atypical Signal Transduction System in Clostridioides difficile
Source: mBio. 2022 Feb 15;13(1):e02969-21. doi: 10.1128/mbio.02969-21 (PMC8844915; doi:10.1128/mbio.02969-21)
Supplement: FIG S5 [file mbio.02969-21-sf005.pdf]

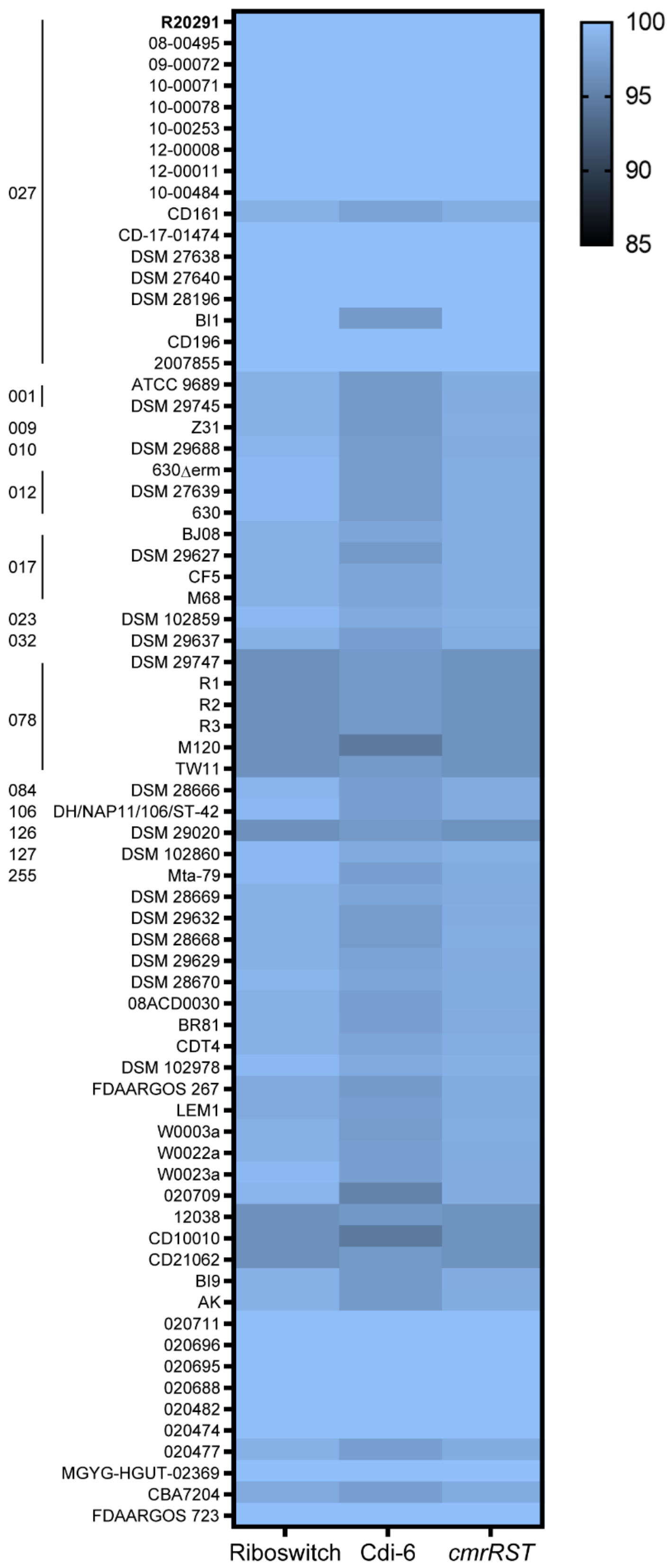

Fig S5. *cmrRST* and upstream regulatory sequences are highly conserved across *C. difficile* strains and ribotypes. Heat map showing percent sequence identity of 71 sequenced *C. difficile* strains in the NCBI database compared to the R20291 reference sequence (NCBI accession number FN545816.1). If the strain has been ribotyped, the ribotype is indicated on the left. For the *cmr* switch, both orientations were used as the reference and the highest percent sequence identity is shown. The following nucleotides were used as the reference sequence from R20291: riboswitch, 3,736,512-3,736,863; *cmr* switch, 3,735,969-3,736,513; *cmrRST*, 3,732,474-3,735,968.
