# Supplementary material for: Multiple Regulatory Mechanisms Control the Production of CmrRST, an Atypical Signal Transduction System in Clostridioides difficile
Source: mBio. 2022 Feb 15;13(1):e02969-21. doi: 10.1128/mbio.02969-21 (PMC8844915; doi:10.1128/mbio.02969-21)
Supplement: TABLE S1 [file mbio.02969-21-st001.pdf]

S1 Table. Strains and plasmids used in this study

Lab Notation	Strain Name	Description	Reference
AC472	*Escherichia coli* DH5α	F- φ80*lacZ*ΔM15 Δ(*lacZYA-argF*)U169 *recA1 endA1 hsdR*17(rκ -, mκ+) *phoA supE44 thi*-1 *gyrA96 relA1* λ- *tonA*	Invitrogen (1)
RT270	*Escherichia coli* HB101(pRK24)	*E. coli* used in conjugations with *C. difficile*, Ap^R, Cm^R	(2)
RT273	*C. difficile* R20291	Ribotype 027 strain, WT (Genbank Accession # FN545816)	(3)
RT2395	R20291 *cmr*-Δ3 OFF	R20291 with the *cmr* invertible element locked in the OFF orientation due to the deletion of three nucleotides in the right inverted repeat	This work
RT2406	R20291 *cmr*-Δ3 ON	R20291 with the *cmr* invertible element locked in the ON orientation due to the deletion of three nucleotides in the right inverted repeat	This work
RT2256	R20291 Δ*cmrR*	R20291 with in-frame deletion of *cmrR*	(4)
RT2296	R20291 Δ*cmrR*Δ*cmrT*	R20291 with in-frame deletions of *cmrR* and *cmrT*	This work
RT2435	R20291 *cmr*-Δ3 ON vector	R20291 *cmr*-ON with pRT1611; vector control	This work
RT2436	R20291 *cmr*-Δ3 ON pP_{tet}::*dccA*	R20291 *cmr*-ON with pRT1587 for inducible *dccA* expression to increase c-di-GMP	This work
RT2437	R20291 *cmr*-Δ3 ON pP_{tet}::EAL	R20291 *cmr*-ON with pRT2444 for inducible *pdcA* EAL domain expression to decrease c-di-GMP	This work
RT2438	R20291 *cmr*-Δ3 OFF vector	R20291 *cmr*-OFF with pRT1611; vector control	This work
RT2439	R20291 *cmr*-Δ3 OFF pP_{tet}::*dccA*	R20291 *cmr*-OFF with pRT1587 for inducible *dccA* expression to increase c-di-GMP	This work
RT2440	R20291 *cmr*-Δ3 OFF pP_{tet}::EAL	R20291 *cmr* locked OFF with pRT2444 for inducible *pdcA* EAL domain expression to decrease c-di-GMP	This work
RT2187	R20291 *cmrR*::SNAP	R20291 with *cmrR* replaced by allelic exchange with a SNAP-tag coding sequence	(5)
RT2500	R20291 *cmrR*::SNAP vector	R20291 *cmrR*::SNAP with pRT1611	This work
RT2501	R20291 *cmrR*::SNAP pP_{tet}::*dccA*	R20291 *cmrR*::SNAP with pRT1587 for inducible *dccA* expression to increase c-di-GMP	This work
RT1693	R20291 *recV cmr-OFF*	R20291 with an insertional mutation in *recV* (*recV::ermB)*; *cmr* locked OFF	(6)
RT2502	R20291 *recV cmr*-OFF pMC123::*phoZ*	R20291 *recV::ermB cmr*-OFF with pMC123::*phoZ* (vector control)	This work
RT2507	R20291 *recV cmr*-OFF pMC123::TSS4-*phoZ*	R20291 *recV::ermB cmr*-OFF with pRT2497 with reporter for TSS4 region only	This work
RT2516	R20291 *recV cmr*-OFF pMC123::*cmr*OFF-*phoZ*	R20291 *recV::ermB cmr*-OFF with pRT2514 with reporter for *cmr*-OFF sequence	This work
RT2517	R20291 *recV cmr*-OFF pMC123:: *cmr*ON-*phoZ*	R20291 *recV::ermB cmr*-OFF with pRT2515 with reporter for *cmr*-ON sequence	This work
RT1615	R20291 vector	R20291 with pRT1611 (vector control)	(7)
RT2085	R20291 pCmrR	R20291 with pRT2073 (P_{tet}::*cmrR*)	(4)
RT2107	R20291 pCmrT	R20291 with pRT2106 (P_{tet}::*cmrT*)	(4)

RT2463	R20291 *cmr*-Δ3 OFF pCmrR	R20291 *cmr* locked OFF with pRT2073 (P_{tet}::*cmrR*)	This work
RT2465	R20291 *cmr*-Δ3 ON pCmrR	R20291 *cmr* locked ON with pRT2106 (P_{tet}::*cmrT*)	This work
RT2269	R20291 Δ*cmrT* vector	R20291 Δ*cmrT* with pRT1611 (vector control)	(4)
RT2402	R20291 Δ*cmrT* pCmrR	R20291 Δ*cmrT* with pRT2073 (P_{tet}::*cmrR*)	This work
RT2270	R20291 Δ*cmrT* pCmrT	R20291 Δ*cmrT* with pRT2106 (P_{tet}::*cmrT*)	(4)
RT2183	R20291 *recV cmr*-OFF pMC-P_{cpr}	R20291 *recV::ermB cmr*-OFF with pMC-P_{cpr}	This work
RT2184	R20291 *recV cmr*-OFF pDccA	R20291 *recV::ermB cmr*-OFF with pMC-P_{cpr}::*dccA*	This work
RT1697	R20291 *recV cmr*-OFF pRecV	R20291 *recV::ermB cmr*-OFF with pP_{tet}-RecV	(8)
RT2520	R20291 *recV cmr*-ON	R20291 *recV::ermB cmr* locked ON, derived from RT1693	This work
RT2198	R20291 *recV cmr*-OFF vector	R20291 *recV::ermB cmr*-OFF with pRT1611 (vector control)	This work
RT2543	R20291 *recV cmr*-OFF pCmrR	R20291 *recV::ermB cmr*-OFF with pRT2073 (P_{tet}::*cmrR*)	This work
RT2544	R20291 *recV cmr*-ON vector	R20291 *recV::ermB cmr*-ON with pRT1611 (vector control)	This work
RT2545	R20291 *recV cmr*-ON pCmrR	R20291 *recV::ermB cmr*-ON with pRT2073 (P_{tet}::*cmrR*)	This work
RT2826	R20291 *recV* CDR2492::P_{tet}::*cmrR*	R20291 *recV::erm* with ATc-inducible *cmrR* integrated between CDR20291_2492 and 2493	This work
RT2827	R20291 *recV* CDR2492::P_{tet}::*cmrR* vector	Inducible *cmrR* strain with pRT1343 (pMC123::*phoZ*)	This work
RT2828	R20291 *recV* CDR2492::P_{tet}::*cmrR* pMC123::TSS4-*phoZ*	Inducible *cmrR* strain with pRT2497	This work
RT2829	R20291 *recV* CDR2492::P_{tet}::*cmrR* pMC123::*cmr*OFF/TSS4-*phoZ*	Inducible *cmrR* strain with pRT2514	This work
RT2830	R20291 *recV* CDR2492::P_{tet}::*cmrR* pMC123::*cmr*ON/TSS4-*phoZ*	Inducible *cmrR* strain with pRT2515	This work
RT2831	R20291 *recV* CDR2492::P_{tet}::*cmrR* pMC123::5'UTR*cmr*OFF-*phoZ*	Inducible *cmrR* strain with pRT2565	This work
RT2832	R20291 *recV* CDR2492::P_{tet}::*cmrR* pMC123::TSS1-*phoZ*	Inducible *cmrR* strain with pRT2566	This work
RT2833	R20291 *recV* CDR2492::P_{tet}::*cmrR* pMC123::*cmr*OFF-*phoZ*	Inducible *cmrR* strain with pRT2567	This work
RT2834	R20291 *recV* CDR2492::P_{tet}::*cmrR* pMC123::*cmr*ON-*phoZ*	Inducible *cmrR* strain with pRT2568	This work
**Lab Notation**	**Plasmid Name**	**Description**	**Reference**
	pMTL-SC7215	Vector for allelic exchange in *C. difficile* R20291	(9)
	pRPF185	*E. coli – C. difficile* shuttle vector, contains ATc-inducible P_{tet} promoter with *gusA*	(10)
	pRT1611	Derivative of pRPF185 with *gusA* removed, vector control	(7)

pRT1587	pP$_{tet}$::*dccA*	pRPF185 with *gusA* replaced by *dccA*, ATc-inducible expression	This work
pRT2444	pP$_{tet}$::EAL	pRPF185 with *gusA* replaced by EAL domain sequence from *pdcA*, ATc-inducible expression	This work
pRT2073	pCmrR	pRPF185 with *gusA* replaced by *cmrR*, ATc-inducible expression	(4)
pRT2106	pCmrT	pRPF185 with *gusA* replaced by *cmrT*, ATc-inducible expression	(4)
	pMC123	*E. coli* – *C. difficile* shuttle vector	(2)
pRT402	pDccA	pMC-P$_{cpr}$::*dccA* (nisin-inducible)	(11)
pRT1343	pMC123::*phoZ*	pMC123 with *Enterococcus faecalis phoZ*	(7)
pRT2497	pMC123::TSS4-*phoZ*	*phoZ* transcriptional reporter of the region between *cmrR* and right inverted repeat of the *cmr* invertible element	This work
pRT2514	pMC123::*cmr*OFF/TSS4-*phoZ*	*phoZ* transcriptional reporter of the region from the *cmr* invertible element (OFF) to *cmrR*	This work
pRT2515	pMC123::*cmr*ON/TSS4-*phoZ*	*phoZ* transcriptional reporter of the region from the *cmr* invertible element (ON) to *cmrR*	This work
pRT2566	pMC123::TSS1-*phoZ*	phoZ transcriptional reporter of the region from the TSS1 promoter/c-di-GMP riboswitch to the LIR of the *cmr* switch	This work
pRT2567	pMC123::*cmr*OFF-*phoZ*	*phoZ* transcriptional reporter of the region from the *cmr* invertible element (OFF) excluding TSS4 region	This work
pRT2568	pMC123::*cmr*ON-*phoZ*	*phoZ* transcriptional reporter of the region from the *cmr* invertible element (ON) excluding TSS4 region	This work
pRT2565	pMC123::5'UTR*cmr*OFF-*phoZ*	*phoZ* transcriptional reporter of the full *cmrRST* regulatory region with *cmr* switch OFF	This work
	pMSR0	Vector for allelic exchange in *C. difficile* R20291, uses toxin-antitoxin counterselection	(12)
pRT2825	pMSR0::CDR2492-P$_{tet}$::*cmrR*-CDR2493	Allelic exchange vector for inserting P$_{tet}$::*cmrR* between CDR20291_2492 and CDR20291_2493	This work

References

1. Hanahan, D. (1983) Studies on transformation of Escherichia coli with plasmids. *J Mol Biol* **166**, 557-580
2. McBride, S. M., and Sonenshein, A. L. (2011) Identification of a genetic locus responsible for antimicrobial peptide resistance in *Clostridium difficile*. *Infection and immunity* **79**, 167-176
3. Stabler, R. A., He, M., Dawson, L., Martin, M., Valiente, E., Corton, C., Lawley, T. D., Sebaihia, M., Quail, M. A., Rose, G., Gerding, D. N., Gibert, M., Popoff, M. R., Parkhill, J., Dougan, G., and Wren, B. W. (2009) Comparative genome and phenotypic analysis of *Clostridium difficile* 027 strains provides insight into the evolution of a hypervirulent bacterium. *Genome biology* **10**, R102
4. Garrett, E. M., Sekulovic, O., Wetzel, D., Jones, J. B., Edwards, A. N., Vargas-Cuebas, G., McBride, S. M., and Tamayo, R. (2019) Phase variation of a signal transduction system controls *Clostridioides difficile* colony morphology, motility, and virulence. *Plos Biology* **17**. e3000379
   10.137/journal.pbio.30003791
5. Sekulovic, O., Mathias Garrett, E., Bourgeois, J., Tamayo, R., Shen, A., and Camilli, A. (2018) Genome-wide detection of conservative site-specific recombination in bacteria. *PLoS Genet* **14**, e1007332
6. Sekulovic, O., Ospina Bedoya, M., Fivian-Hughes, A. S., Fairweather, N. F., and Fortier, L. C. (2015) The *Clostridium difficile* cell wall protein CwpV confers phase-variable phage resistance. *Molecular microbiology* **98**, 329-342
7. Anjuwon-Foster, B. R., and Tamayo, R. (2017) A genetic switch controls the production of flagella and toxins in *Clostridium difficile*. *PLoS Genet* **13**, e1006701
8. Anjuwon-Foster, B. R., and Tamayo, R. (2018) Phase variation of *Clostridium difficile* virulence factors. *Gut Microbes* **9**, 76-83
9. Cartman, S. T., Kelly, M. L., Heeg, D., Heap, J. T., and Minton, N. P. (2012) Precise manipulation of the *Clostridium difficile* chromosome reveals a lack of association between the *tcdC* genotype and toxin production. *Applied and Environmental Microbiology* **78**, 4683-4690
10. Fagan, R. P., and Fairweather, N. F. (2011) *Clostridium difficile* has two parallel and essential Sec secretion systems. *The Journal of biological chemistry* **286**, 27483-27493
11. Purcell, E. B., McKee, R. W., McBride, S. M., Waters, C. M., and Tamayo, R. (2012) Cyclic diguanylate inversely regulates motility and aggregation in *Clostridium difficile*. *Journal of Bacteriology* **194**, 3307-3316
12. Peltier, J., Hamiot, A., Garneau, J. R., Boudry, P., Maikova, A., Hajnsdorf, E., Fortier, L. C., Dupuy, B., and Soutourina, O. (2020) Type I toxin-antitoxin systems contribute to the maintenance of mobile genetic elements in Clostridioides difficile. *Commun Biol* **3.**
    10.1038/s42003-020-01448-
