# Supplementary material for: Multiple Regulatory Mechanisms Control the Production of CmrRST, an Atypical Signal Transduction System in Clostridioides difficile
Source: mBio. 2022 Feb 15;13(1):e02969-21. doi: 10.1128/mbio.02969-21 (PMC8844915; doi:10.1128/mbio.02969-21)
Supplement: TABLE S2 [file mbio.02969-21-st002.pdf]

S2 Table. Primers used in this study.

Primer Name	Primer Sequence (5' to 3')
OS383	GTTTTTTGTTACCCTAAGTTTAGTAATAGTATCAAGAGAAGAAGG
OS384	AGTCTCCGGTTGGAGAATGGAGCTTAAAG
OS385	TCTCCAACCGGAGACTTTAATTAGTATTTAATAATAAATC
OS386	GATTATCAAAAAGGAGTTTCCATTACTTGCTCTAATAGAC
OS268	TTTTTTGTTACCCTAAGTTTGCCATCCATGTATCCTATC
OS269	TTAATATATTTATATAATCACTCCCAAATATCATTATTC
OS271	AGATTATCAAAAAGGAGTTTTGTCAAATCTTGTAACCAAC
OS283	AGTGATTATAGAAAATTATAACAATAAGAGGAGC
OS387	GACTTTAAGCTCCATTCTCCAACAAT
OS137	GAACAATTCTTGAATATTGTATTGAACATTAAGA
OS109	GGAGATATATGGAGTTAGTGGTGCAA
OS111	CGCTCTACTATATCCATAGCATCTTT
OS388	AGTCTCCGGTTGGAAAAGGGAAATTTTTTAAAAAG
OS389	TTTTCCAACCGGAGACTTTAATTAGTATTTAATAATAAATC
OS390	CTTTTTAAAAAATTTCCCTTTTCCAACAAT
OS391	CACCACTCCATTCAAAGGTATTTTAATC
R1907	CAGAGCTCCTAGTACAAAGTATTTTATTTTGGAG
R1908	GACGGATCCCAGTACTTAATGTCAATATCTTGTATAG
R2689	CAGAGCTCCTATGGAGGAGATAAGTATATGGATTTAAAGGCATCAATTAATGATG
R2690	CAGGATCCATACTATTCCCAATTTAACATCC
R2754	ATGCAGAATTCACTTTAATTAGTATTTAATAATAAATCTTAATG
R2342	ATGCAGGATCCCATTACACCACTCCATTCAAAGG
R2337	ATGCAGAATTCGGTGAAATTTTGGCTTTTAAAGTAGCC
R2270	GGAGATATATGGAGTTAGTGGTGCAA
R2271	CTAGCCAATAGACAAGTTTCTAGAAAAATA
R2272	GAACAATTCTTGAATATTGTATTGAACATTAAGA
R850	CTAGCTGCTCCTATGTCTCACATC
R851	CCAGTCTCTCCTGGATCAACTA
R2918	CTCCTGAAATACTTGATGAACCAGAAGCTTAAGACCCACTTTCACATTTAAGTTG
R2919	GGTATATGATATGAAAGAGAGAGTCTCAAACAGTATCTCACTTATGGTACAAACTTATATCC
R2914	CATTGATTTCTTTCAGTTTCGGATCCCTCCTGAAATACTTGATGAACCAGAAG
R2915	CAACTTAAATGTGAAAGTGGGTCTTAAGGTGAACTTTTGTTCATGAGACACTC
R2916	GGATATAAGTTTGTACCATAAGTGAGATACTGTTTGAGACTCTCTCTTTCATATCATATACC
R2917	GACGTCGACTCTAGAGGATCCTACATGCAATAACAGTACCTCAGGA
R2743	GTGTTATCAATTGCACTACTCATGG
R2744	GTTGAACCATTAGCTAAGGATTCAG
R2987	GTATGTTAGAAGTTGTTTCAGAAGGC
R2988	GTGGCTGAAGTAGTATCAGAAGC
R2298	AAGAAAAAGTTCGGGGATTTTTAGC
R2299	CGCTGAAAACTTTAACACATTAGGA
R2537	GACAAGGATAATTGCC
R2538	CCATCACCATCAGTTAG

R2539	GATAGATGACTGGGAG
R2540	CGATAAGTAGCATTCCC
R2745	CTTTTTTTGCTTTAAAATTAACAAAAATGTTGC
R2746	CTTTAAAAGCCAAAATTTCACCTATCAATA
R2751	CTTAATGTTCAATACAATATTCAAGAATTG
R2803	GAAAATAAAACATTATAATTTGTAATAATTATAAAACTTGAG
R2804	CCAGTAAATTATACATTACACCACTCC
R2716	GTCTCTGATGTATATACATTAAAACC
R2273	TCATTACCAGGTGTAGCAGTGAATGC
R2274	GATAGAGCATGGTCCTTGAGCTTCT
R2792	GTCTAAAACTATACGCTC
R2793	CCATAGCATCTTTAGCAGTCTCTGATGTATATA
R3171	ACCATATAAATCATATAAATC
R3172	ACTCAAACTAGCCAATAGACAAGTTTCTAG

Restriction sites are underlined. Regions of homology for Gibson assembly are lowercase.
